# A Person-Based Web-Based Sleep Intervention Aimed at Adolescents (SleepWise): Randomized Controlled Feasibility Study

**DOI:** 10.2196/51322

**Published:** 2024-10-23

**Authors:** Shokraneh Moghadam, Margaret Husted, Ana Aznar, Debra Gray

**Affiliations:** 1 Department of Psychology University of Winchester Winchester United Kingdom

**Keywords:** web-based health interventions, sleep, adolescence, behavior change, person-based approach, sleep intervention, detrimental health outcome, SleepWise

## Abstract

**Background:**

Adolescents are advised to sleep 8-10 hours per night; however, most do not sleep for this recommended amount. Poor adolescent sleep is associated with detrimental health outcomes, including reduced physical activity, risk-taking behaviors, and increased depression and anxiety levels, making this an important public health concern. Existing interventions targeting adolescent sleep are often unsuccessful or their effectiveness unclear, as they are frequently noninteractive, time-consuming, and lack a strong theoretical foundation; highlighting an urgent need for innovative interventions deemed acceptable by adolescents.

**Objective:**

The main objective of this study was to determine the acceptability, feasibility, and preliminary impact of a web-based person-based sleep intervention (SleepWise) on adolescent sleep quality. Participant incentivization was also explored to understand its impact on engagement, acceptability, and sleep quality.

**Methods:**

A feasibility trial was conducted to test the feasibility, acceptability, and preliminary impact of SleepWise on adolescent sleep quality, developed based on the person-based approach to intervention development. In total, 90 participants (aged 13-17 years) from further education institutions and secondary schools were recruited for two 2-arm randomized controlled trials. One trial (trial 1) was incentivized to understand the impact of incentivization. Acceptability and sleep quality were assessed via questionnaires, and a mixed methods process evaluation was undertaken to assess participant engagement and experience with SleepWise. Engagement was automatically tracked by SleepWise, which collected data on the date and time, pages viewed, and the number of goals and sleep logs completed per participant. Semistructured interviews were carried out to gain participant feedback.

**Results:**

Participants in both trials reported high levels of acceptability (trial 1: mean 21.00, SD 2.74; trial 2: mean 20.82, SD 2.48) and demonstrated similar levels of engagement with SleepWise. Participants in trial 1 viewed slightly more pages of the intervention, and those in trial 2 achieved their set goals more frequently. Improvements in sleep quality were found in both trials 1 and 2, with medium (trial 1) and large (trial 2) effect sizes. A larger effect size for improvement in sleep quality was found in the nonincentivized trial (*d*=0.87), suggesting that incentivization may not impact engagement or sleep quality. Both trials achieved acceptable recruitment (trial 1, N=48; trial 2, N=42), and retention at 5 weeks (trial 1: N=30; trial 2: N=30). Qualitative findings showed that adolescents lead busy lifestyles, which may hinder engagement; however, participants deemed SleepWise acceptable in length and content, and made attempts at behavior change.

**Conclusions:**

SleepWise is an acceptable and potentially efficacious web-based sleep intervention aimed at adolescents. Findings from this study showed that incentivization did not greatly impact engagement, acceptability, or sleep quality. Subject to a full trial, SleepWise has the potential to address the urgent need for innovative, personalized, and acceptable sleep interventions for adolescents.

**Trial Registration:**

OSF Registries osf.io/yanb2; https://osf.io/yanb2

## Introduction

### Background

Poor sleep quality among adolescents is an important public health problem [1,2]. Research shows that 53% of adolescents get less than 8-hours of sleep per night and do not sleep for the recommended amount of 8-10 hours, especially on school nights [3-6]. Additionally, at least 36% of adolescents experience difficulty falling asleep, with 59% waking up feeling tired during the week [3-6]. Poor sleep quality in adolescents is associated with detrimental health outcomes, including unhealthy weight gain, reduced levels of physical activity, increased risk-taking behaviors, and increased levels of anxiety and depression, which could risk continuation into adulthood [7-13].

In recognition of the clear public health implications of poor adolescent sleep, there are several interventions aimed at improving sleep quality in adolescents [13]. These include school-based sleep education programs, which have been shown to successfully improve sleep knowledge in adolescents, but to be less useful for improving sleep behavior, as information is rarely enough to change behavior or guarantee positive sleep outcomes [14,15]. Interventions aimed at targeting specific behaviors, such as reducing screen time before sleep, have proven somewhat more successful for improving sleep duration and sleep-related behaviors among adolescents [16]. Likewise, interventions that include cognitive behavior therapy (CBT) have been found to be successful, producing significant improvements in both objective and subjective sleep outcomes among adolescents [17].

Determining the success of sleep interventions is difficult, as research in this area is often limited by a lack of control groups (CGs) and the application of a strong theoretical foundation, which makes it difficult to determine the mechanisms that make an intervention effective. Moreover, these interventions are often viewed as undesirable by adolescents due to being noninteractive and time-consuming [14,18-20]. Coupled with these shortcomings, sleep interventions targeted at adolescents can also be limited by small sample sizes, high dropout rates, little to no follow-up data, and being costly [13,17,21,22].

There is a pressing need for innovative solutions in this field of research. One clear recommendation is for intervention developers to utilize digital platforms [13,14]. There has already been some success in this field. For example, data suggests that the effects of digitalized CBT programs for insomnia are similar to those for face-to-face programs, indicating improvements in insomnia severity, sleep efficiency, subjective sleep quality, wake after sleep onset, sleep onset latency, total sleep time, and number of nocturnal awakenings [23]. These effects were comparable to those found with face-to-face CBT for insomnia (CBT-I) programs and were commonly maintained at 4- to 48-week follow-up [23]. However, these results were found in an adult population, and research is not as well established in the target population. Nonetheless, in a randomized-controlled trial, de Bruin et al [24] concluded that internet-based and face-to-face CBT-I result in long-term improvements in adolescent affective and anxiety problems by reducing insomnia symptoms, with adolescents preferring digital over face-to-face sleep treatment [25]. Therefore, this supports the use of digital methods of intervention delivery and aligns with adolescents’ preferences for sleep treatment. Evidence suggests that over 90% of adolescents in high-income countries, such as the United Kingdom, have access to the internet [26]. With digital platforms offering an attractive, feasible, and effective method to health management, including sleep behaviors among adolescents [20], there is an urgent demand for innovative, behavioral, and personalized approaches to treating adolescent sleep problems [13]—one that includes the adolescent voice in the development process—such that any intervention that is developed is acceptable to its intended end-users: the adolescents.

### This Study

This study aims to overcome the limitations of previous sleep interventions targeted at adolescents, by undertaking a feasibility trial to assess the feasibility and preliminary impact of an evidence- and theory-based web-based sleep intervention called SleepWise. Specifically, the study adopted the person-based approach (PBA) [27] and the guidelines on the development of complex interventions [28]. The purpose of this feasibility study is to (1) explore recruitment rates, attrition, and uptake of the intervention, and its preliminary impact on sleep quality; (2) conduct a mixed methods process evaluation to determine participant engagement and experience with the intervention; and (3) understand the impact of incentivization on engagement, acceptability, and sleep quality, as the use of incentives needs careful consideration and understanding in research [29]. To investigate the third aim, 2 separate 2-arm randomized controlled feasibility trials were undertaken as part of this study; trial 1 was incentivized, and trial 2 was not.

### The SleepWise Intervention

SleepWise was uniquely developed using the PBA to intervention development [27] by integrating evidence-based behavioral techniques (eg, sleep education) and user feedback during the development process. The PBA combines participatory, theory-based, and evidence-based approaches to intervention development, with the aim of optimizing health interventions by grounding them within the perspectives of the people who use them [27]. While the PBA has been successfully used in previous research [30], it has not been used in this context. The development and optimization of SleepWise, which included addressing adolescent feedback based on a prototype version of the intervention, is reported in full elsewhere. In short, the optimized version of SleepWise reported in this paper consists of 4 weekly web-based sessions, completed over a 4-week period. Each session is tunneled (ie, participants work through a set number of compulsory or main pages), while “click-through” options provide optional and additional information. Session 1 provides information about the importance of sleep for young people and how eating habits can impact sleep. Session 2 contains information about the relationship between physical activity and sleep quality, and sessions 3 and 4 provide information about the importance and relationship between sleep and everyday habits among young people (eg, screen use, sleep environment, etc). Each session takes approximately 15-25 minutes to complete, depending on how much time participants want to spend on each session (eg, exploring optional pages). Participants in this study were encouraged to use SleepWise to set weekly goals and complete a daily sleep diary. At the start of each weekly session, participants reviewed their goals from the previous week and were given the option to set new goals or continue with the same goals. At the end of each weekly session, participants had the option to complete quizzes and take part in educational and interactive games (see Multimedia Appendices 1 and 2). On the SleepWise website, participants were also provided with optional sleep-aiding tools such as sleep routine charts and mindfulness recordings.

## Methods

### Overview

Two separate randomized controlled feasibility trials were undertaken as part of this study (OSF registration ID https://osf.io/yanb2). The only difference between the trials was that trial 1 was incentivized and trial 2 was not. This was to determine the impact of incentivization as part of the feasibility study. Trial 1 recruitment took place from November 2018 to May 2019 (study running in the summer term), with a rolling start time. Trial 2 recruitment took place from May 2019 to December 2019 (study running in the autumn term), also with a rolling start time.

### Recruitment

Participants were recruited from 3 secondary schools and 2 further education colleges in Southeast England. The eligibility criteria for all parts of the study included being aged between 13 and 19 years and having access to a computer or laptop with internet access. Participants were not screened for any sleep, mental, or physical health conditions, as SleepWise is intended for use in a general population of adolescents, and not in an exclusive clinical or sub-clinical group.

Participant recruitment happened via the primary researcher reaching out to schools and colleges advertising the study and sending posters to those who responded as interested. An allocated member of staff (study facilitator) at each study site (schools and colleges) displayed or circulated the poster advertising the study to participants. Sites were offered a short talk advertising the study; if they chose this option, the researcher played a short video about adolescent health and talked about the aims of the study.

### Ethical Considerations

Ethical approval for the study was received from the university ethics committee (RKEEC190204), and the study was undertaken in accordance with the Helsinki Declaration. Adolescents who were interested in partaking in the study collected consent and information sheets from the study facilitator or by emailing the primary researcher. Signed consent forms were then collected by the primary researcher from each site or emailed directly to the researcher by participants. Parental consent was not required for participants aged 16 years and over, as per the British Psychological Society’s ethical guidelines [31].

This study maintained the confidentiality and anonymity of study participants. Study-related data were maintained on a password-protected computer, and any paper-based records were kept in a locked university cabinet. Participant data were anonymized. Intervention participants in trial 1 received a £20 (US $26.16) voucher once follow-up questionnaires were completed. This incentive was only offered to participants in the intervention group (IG) who undertook the intervention and completed the 5-week follow-up questionnaires in trial 1. All participants who took part in the qualitative interviews as part of the (poststudy) process evaluation received a £20 (US $26.16) voucher as a nominal payment for their time.

### Procedure

Participants in each trial were randomly allocated to an intervention or CG using an internet-based randomization generator (Randomisation.com). Participants were not blinded to allocation due to challenges associated with blinding participants in web-based trials [32,33]. The primary researcher was the intervention administrator, assessor, and data analyzer, and therefore it was not possible to blind them. Allocation was revealed to participants in an email containing information on how to access the intervention (IG), or to continue as normal (CG). Control participants continued to receive standard education (eg, at school) but were informed about the importance of their role in the study and were given the option to access the intervention (at the end of the trial) if they wished. After randomization, participants completed a baseline measure questionnaire about their sleep quality via an internet-based survey (Qualtrics). Participants in both groups were asked to complete this measure at baseline and at 5-week follow-up. Intervention participants were asked additional questions about the intervention’s acceptability at the 5-week stage. Text and email reminders were sent to participants if they had not responded to follow-up (5-week) questionnaires; however, participants had the option to opt out of these reminders if they wished. Participants in the IG were emailed weekly links to access the (free of charge) intervention and, with instructions, were asked to work through it for 4-5 weeks. Intervention participants were also sent weekly email and text reminders, reminding them about their goals and sleep logs. Participants also had the option to opt out of these reminders if they wished.

Regarding incentivization, intervention participants in trial 1 received a £20 (US $26.16) voucher once follow-up (5-week) questionnaires were complete. This incentive was only offered to participants in the IG who undertook the intervention and completed the 5-week follow-up questionnaires in trial 1. Participants in trial 2 did not receive incentives.

### Process Evaluation

The aim of process evaluations is to explore implementation and contextual outcomes within a trial, which helps to better understand how best to improve interventions and their delivery [34]. The process evaluation in this study consisted of both quantitative and qualitative data collection and analysis. Specifically, to better understand participant engagement with the intervention, the quantitative component of the process evaluation evaluated participants’ engagement with each weekly session (date and time and pages viewed) and the number of goals and sleep logs completed across the intervention period, using SleepWise’s automatic data tracker. The qualitative component of the process evaluation explored participants’ experiences with SleepWise via qualitative follow-up interviews. The findings from these interviews can help inform the implementation of the intervention in a future trial of effectiveness. Participants from both trials in the intervention and CGs were contacted between 5 and 8 weeks post study via telephone interviews. Participants received a nominal £20 (US $26.16) voucher on completion of qualitative process evaluation interviews. This participant payment was separate from the incentivization evaluation, as the intervention period had ended.

### Measures

#### The Pittsburgh Sleep Quality Index–Short Form

The Pittsburgh Sleep Quality Index–Short Form (PSQI-SF) [35] was used to measure sleep quality among participants. The PSQI-SF is a well-validated self-rated questionnaire that has been widely used in the adolescent population to assess subjective sleep quality and disturbances and the impact of poor sleep on functioning [36,37]. The PSQI-SF contains 13 questions and measures 5 dimensions: sleep latency, sleep duration, sleep efficiency, sleep disturbances, and daytime dysfunction. Items are rated from 0 (very good) to 3 (very bad) [35]. A total score greater than “4” is indicative of poor sleep quality.

#### Acceptability E-Scale

The acceptability of the intervention was assessed using the 6-item Acceptability E-Scale [38]. This scale measures the extent to which adolescents deem the intervention acceptable. Items are rated from 1 (negative evaluation) to 5 (positive evaluation). Scores on each subscale were summed for a total acceptability score. Higher scores indicate a higher level of acceptance. This measure was slightly adapted (replacing “computer program” with “this website” and removing one item that was not relevant to this study (asked how helpful the program was for describing symptoms and quality of life)).

### Analysis Plan

#### Quantitative Data

Descriptive statistics were used to illustrate participant characteristics. An exploratory effect size analysis was undertaken to explore the initial indicators of SleepWise on the primary outcome of sleep quality. An exploratory effect size, using Cohen *d*, was calculated for the mean changes in sleep quality (PSQI-SF) for intervention participants by subtracting participants’ follow-up sleep quality mean from their baseline sleep quality mean (mean difference) in the 2 study groups by using the SPSS software (version 26; IBM Corp). Cohen *d* is deemed more suitable for determining the effect of an intervention in contrast to *r*, which is more appropriate for evaluating correlations between variables [39]. As noted earlier, data about the date and time, frequency of participants’ weekly goals, sleep logs, and pages visited was automatically tracked by the SleepWise website and used as a measure of participant engagement with the intervention.

#### Qualitative Data

In total, 19 participants (mean age 14.79, SD 0.98 years) were interviewed as part of the process evaluation (see Table 1 for demographics). Interviews were audio recorded and lasted for approximately 20-30 minutes. Interviews stopped when saturation was reached (ie, no new themes were derived from the latest interview transcripts) [40]. The recordings were transcribed verbatim, and an inductive thematic analysis was conducted using Braun and Clarke’s [41] 6 phases to thematic analysis.

**Table 1:** Participant characteristics from the poststudy process evaluation interviews^a^.

Demographics (N=19)^b^		Intervention (n=15)^c^	Control (n=4)^c^
**Gender, n (%)**			
	Male	4 (26.7%)	—^d^
	Female	11 (73.3%)	4 (100%)
**Ethnicity, n (%)**			
	White	13 (86.7%)	4 (100%)
	Indian	2 (13.3%)	—

^a^ Conducted using Braun and Clarke’s [41] 6 phases to thematic analysis.

^b^N: total number of participants from both trials.

^c^n: total sample number.

^d^Not applicable.

## Results

### Quantitative Findings

#### Participant Recruitment and Attrition

**Figure 1:**
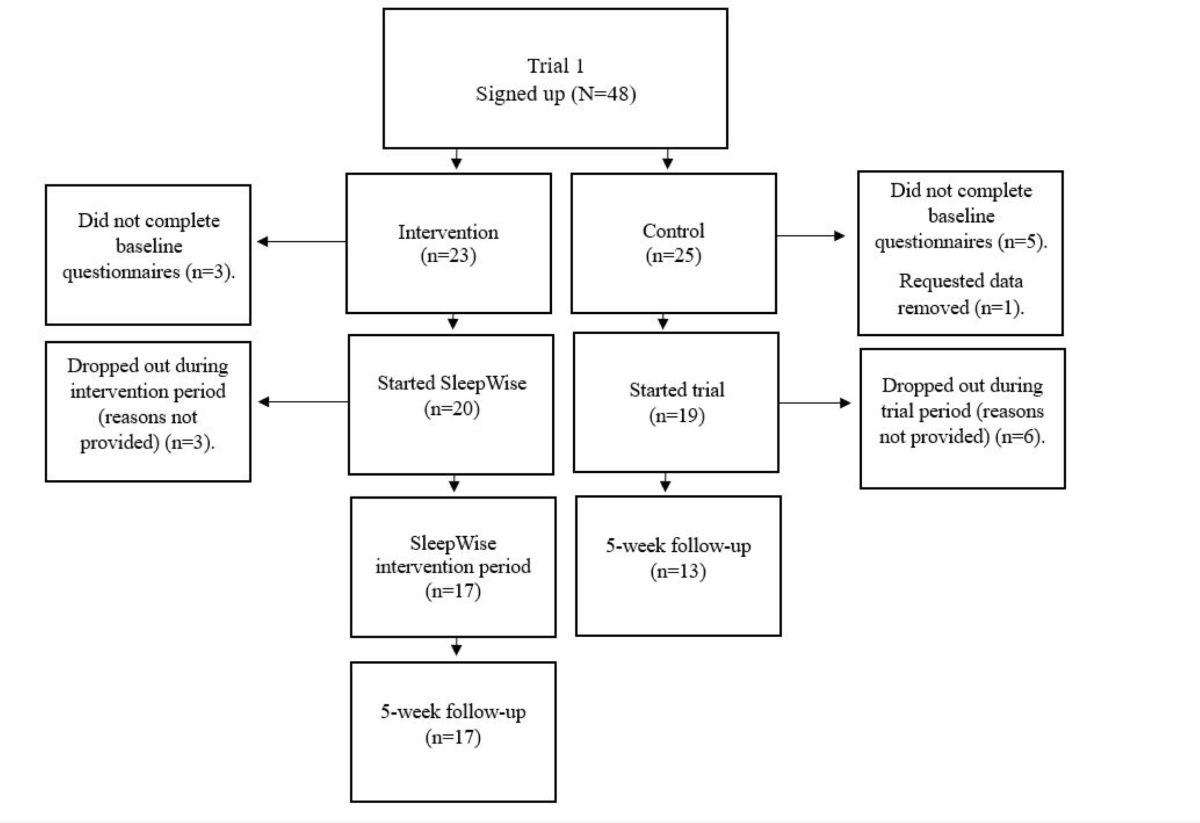
Flowchart of participant recruitment and attrition for trial 1.

**Figure 2:**
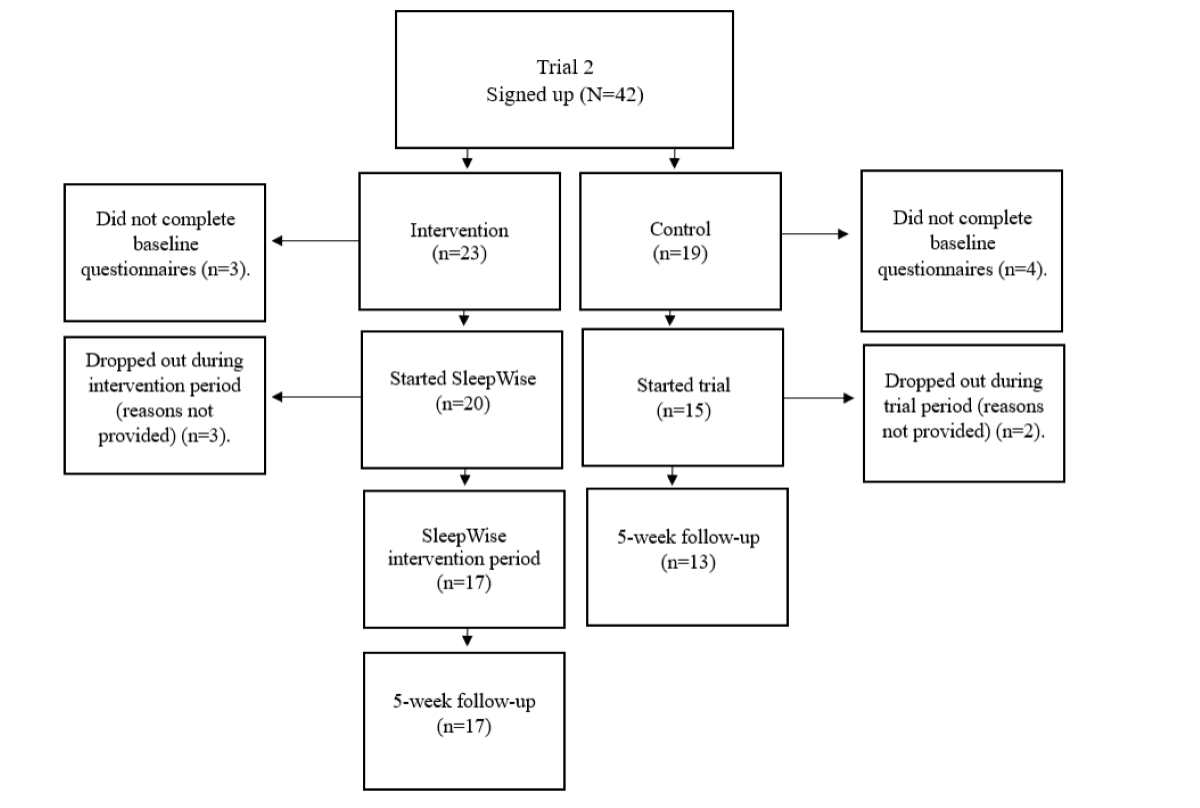
Flowchart of participant recruitment and attrition for trial 2.

Table 2 shows participant characteristics for all participants (in both intervention and control arms, across both trials) who started each trial post randomization, and Figures 1 and 2 show the recruitment and attrition processes in trials 1 and 2. A total of 100 adolescents (50 participants per trial) aged between 13 and 19 years were intended for recruitment. A prior power analysis suggested a participant sample of 100 was required based on the assumption that it allowed for a 40%-50% dropout or loss to follow-up, which is common in internet-based research [42]. This resulted in an overall sample of at least 50 participants per trial (25 for intervention and 25 for control). The trials achieved to recruit 90 participants in total and dropout figures were low (Figures 1 and 2).

**Table 2:** Participant characteristics (intervention and control) at the start of each trial post randomization.

Characteristics		Trials		
		Trial 1 (N=39)		Trial 2 (N=35)
Age, mean (SD); median (IQR)		14.74 (0.44); 15.0 (14-15)		14.71 (1.07); 14.0 (14-17)
**Gender,** **n** **(%)**				
	Male	11 (28.2)		6 (17.1)
	Female	26 (66.7)		29 (82.9)
	Non-binary	1 (2.6)		—^a^
	Transgender male	1 (2.6)		—
**Ethnicity,** **n** **(%)**				
	White	34 (87.2)		32 (91.4)
	Chinese or South East Asian	—	—	
	Black African	—	—	
	Indian	2 (5.1)		2 (5.7)
	Black Caribbean	—	—	
	Pakistani	—	—	
	Bangladeshi	—	—	
	Other—Black British	1 (2.6)		—
	Other—mixed heritage	2 (5.1)		—
	Other—native American	—		1 (2.9)
**School and college year, n (%)**				
	Secondary school: year 10	39 (100)		29 (82.9)
	College: first-year college	—		2 (5.7)
	College: second-year college	—		4 (11.4)

^a^Not applicable.

#### Participant Engagement With SleepWise

##### Number of Pages Viewed

**Figure 3:**
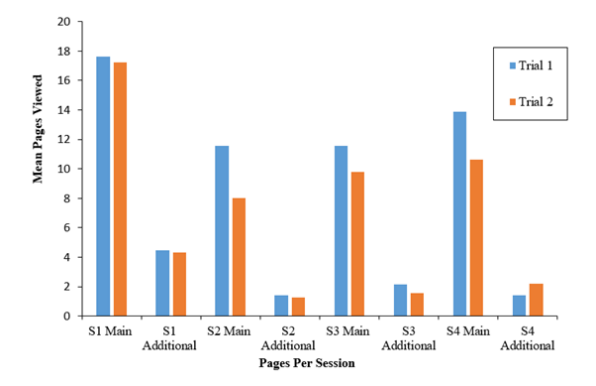
Pages of the intervention viewed per session (S) in each trial.

Participants in both trials showed similar usage patterns, with those in trial 1 (incentivized) showing a higher number of main pages (compulsory website pages) viewed across all sessions (Figure 3). Participants in both trials viewed more pages in the first session (eating and sleeping) than any other session on SleepWise. Participants also explored the additional (optional) pages of the first session more than those in any other session (Figure 3), suggesting that adolescents may be more interested and therefore more inclined to spend time on sessions that contain information about the importance of sleep and the impact of eating behaviors on sleep. Nevertheless, high engagement with session 1 could also be explained by the fact that session 1 was the first intervention session, and thus the novelty of the intervention may have encouraged participants to explore the first session more than subsequent sessions. Nonetheless, engagement was also high for session 4 (sleep and everyday habits and environments), and while this was slightly higher in trial 1, these results may suggest that topics specifically related to improving sleep habits, such as sleep-related eating behaviors (session 1) or everyday habits (session 4), encourage higher levels of engagement.

##### Frequency of Sleep Logs, Goals, and Goal Reviews

**Figure 4:**
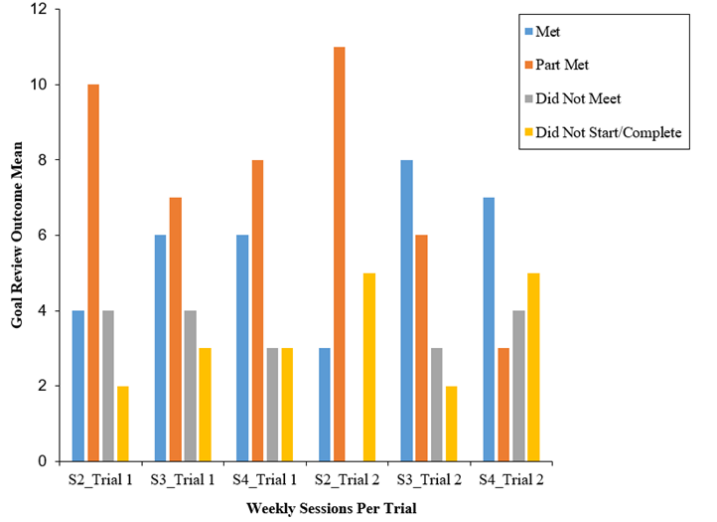
Goal reviews per intervention session (S) in each trial.

Across both trials, participants completed a similar mean total of sleep logs and logged goals across the intervention sessions (trial 1 sleep log mean 8.25, goals mean 2.0; trial 2 sleep log mean 8.58, goals mean 2.05). However, participants in trial 2 (nonincentivized) “met” their goals more frequently than those in trial 1 (Figure 4). Participants in trial 1 mostly “part met” their goals, which may tentatively suggest that participants in trial 2 were more successful in achieving their goals than those in trial 1. While these findings may overall suggest that participants in the incentivized trial (trial 1) showed slightly higher engagement levels across all sessions (Figure 3), those in the nonincentivized trial (trial 2) “met” their goals more frequently, suggesting that incentivization may not necessarily encourage higher levels of engagement or behavior change (such as achieving set goals) among adolescents.

#### Exploratory (Sleep Quality) Effect Sizes and Intervention Acceptability in Trials 1 and 2

##### Effect Size

An exploratory effect size study was undertaken for both trials to investigate whether SleepWise and incentivization had any initial impacts on sleep quality (effect size) scores. Effect sizes were interpreted using the following criteria: <0.30 small effect size; 0.30-0.80 medium effect size; and >0.80 large effect size [43].

##### Trial 1—Incentivized

Based on the 5-week post-study follow-up sleep quality data (PSQI summed score) from 17 participants, the IG improved sleep quality by an average (PSQI-SF) mean of 1.35 (SD 2.12, 95% CI 0.263-2.443). Participants in the CG (n=13) improved sleep quality by an average mean of 0.23 (SD 1.83, 95% CI –0.877 to 1.338). A medium effect size was found based on participants’ mean change in sleep quality between the 2 study groups (Cohen *d*=0.57). This medium effect size suggests that the IG’s sleep quality scores improved more than those in the CG. This suggests that the intervention is likely to improve sleep quality scores.

##### Trial 2—Nonincentivized

Based on the 5-week post-study follow-up sleep quality data from 17 participants, the IG improved sleep quality by an average (PSQI) mean of 0.94 (SD 1.44, 95% CI 0.203-1.679). Participants in the CG (n=13) showed reduced sleep quality by an average mean of –0.31 (SD 1.44, 95% CI –1.176 to 0.560). A large effect size was found based on participants’ mean change in sleep quality between the 2 study groups, Cohen *d*=0.87. This large effect size suggests that the IG’s sleep quality scores improved more than those in the CG, as the CG’s sleep quality scores worsened over time. Therefore, as with trial 1, it can be concluded that the intervention is likely to improve sleep quality scores in a nonincentivized trial.

##### Acceptability

Mean acceptability scores indicated that participants found the intervention acceptable in both trials (trial 1: mean 21, SD 2.74; trial 2: mean 20.82, SD 2.48). This finding suggests that the intervention could be deemed acceptable by target users and supports the intervention’s feasibility for a future definitive trial.

### Qualitative Findings

In total, 19 participants (mean age 14.79, SD 0.98 years) were interviewed as part of the process evaluation interviews (see Table 1). Interviews lasted for approximately 20-30 minutes, depending on how much information participants provided. After conducting a thematic analysis, 3 main themes were identified: busy lifestyles, self-monitoring: recording sleep and setting goals, and changing behavior. These findings explore participants’ experiences with the intervention and can inform the implementation of the intervention in a future randomized controlled trial. Participant number (P, followed by number), gender, study group (IG and CG), are provided after each quote for informative purposes.

#### Busy Lifestyles

Participants reported leading a busy lifestyle as one of the main barriers to engaging with SleepWise. Academic commitments and the expectation to complete large volumes of homework shifted participants’ sleep patterns to later in the evening: “There is a lot of work, because we are doing our GCSEs now, and so it’s busy, and because we’ve got a lot of homework, we have to stay up really late” [P8, male, IG]. Prioritizing homework often meant that adolescents only completed the required and compulsory activities on SleepWise, and were limited on time to explore additional pages and resources: “I just got really busy [with homework]—I just went on there to do the necessities, and do what I needed to do, and then get on with my homework” [P7, female, IG].

Similarly, leading a busy lifestyle also hindered engagement with other sleep-related tasks, such as logging sleep: “…sometimes I didn’t have time [to log ‘wake-up’ time] because I was rushing and getting ready for school” [P16, female, IG]. However, participants reported that working through the intervention at weekly intervals was a positive delivery feature of the intervention: “…[I] liked how short it was—like I was expecting a lot more stuff I had to do - but I quite liked, you could finish one week’s worth of stuff in like…five minutes” [P4, male, IG], suggesting that SleepWise is time-efficient and well-suited to adolescents’ busy lifestyles.

#### Self-Monitoring: Recording Sleep and Setting Goals

Participants who monitored their sleep using the sleep diary function of the intervention found it interesting to observer their sleep patterns in detail: “[I] liked tracking the sleep, I thought it was quite interesting just to see the data basically, and I hadn’t really looked at much [of my] sleeping pattern in that much detail before” [P2, male, IG]. However, participants reported that the sleep diary could be better accessed on a mobile phone, suggesting that a more accessible and flexible approach to logging sleep may be preferred:

I think it’d be a lot better to fill out the [SleepWise] sleep log on an app rather than a laptop or website because I don’t really go on my laptop until after school and by then I probably forgotten what time I fell asleep [the night before] or woke up [that day].

P4, male, IG

Similar to the weekly session intervals being deemed a time-efficient delivery feature of the intervention, setting goals on a weekly basis also suited adolescents’ busy schedules:

[I] think that four to five weeks was better for me because it meant that you could focus on one goal each week instead of like everything at once, because I don’t think I would’ve taken in much as I did if it was all at once.

P5, female, IG

In addition, goal reviewing was reported as a useful feature for helping participants reflect on and be reminded about their weekly goals:

…it [goal review] helped me reflect…like what I should remember to do, like sometimes, you know, you forget to do stuff, and by the end of the week you get to review your goal, it’s like “yeah I was meant to do that”, and if it didn’t work out, I could replace it with another like goal, like, a healthy ambition.

P3, female, IG

While setting and reviewing goals were mainly reported as positive features of the intervention, participants reported needing more personalization and flexibility over their use of these features. As such, participants suggested a number of improvements. Firstly, when reviewing their goals, participants preferred being asked whether they had (or had not) set a weekly goal in the first instant: “Like a simple programming thing of checking whether I actually created a goal before asking me whether I met it or not” [P4, male, IG]. Participants also reported that they should be able to review their goals independently of the other goals—that is, to be able to indicate whether they met, part met, or not met their goals individually—as opposed to reviewing all goals together: “I think for some weeks, I set more than one goal and I think if I could say if I had part met that goal, and if I fully met another one that’d be useful, not just overall” [P12, female, IG].

Participants suggested that the goal-setting feature should be more personalized, by including personalized goal suggestions (ie, alternative goals) for each user: “…maybe specific to each goal you set, SleepWise would tell you an alternative to set” [P11, female, IG].

#### Changing Behavior

Participants reported that the first session of the intervention included the most interesting and informative topics: “I just liked looking through how food affects your sleep because I hadn’t heard that before, so I just thought it was interesting to read up [on it]” [P7, female, IG]. For example, understanding the impact of eating habits on sleep: “SleepWise said that if you carry on snacking on unhealthy foods, it can cause disruption in your sleep, and chemical imbalances in your body, so I just thought, well, that’s not very good” [P7, female, IG].

While session 1 was deemed informative and interesting, participants were also drawn to and engaged with subsequent sessions on sleep (sessions 3-4), attempting behavioral changes such as reducing screen time before sleep: “I was trying to put my phone down before I go to bed because blue light is really bad for your brain” [P3, female, IG], and keeping to a consistent bedtime routine: “I tried to keep my bedtime consistent the entire time, and that helped keep the time I woke up quite consistent so I usually got the same amount of sleep every night” [P11, female, IG]. However, participants’ attempts at behavior change were encouraged by how relatable they found the content of each session:

I think it [Session 3] was just… relatable on my part, because I do go on my kindle, or watch television, before I actually go to sleep, and so I think that’s why I spent [more] time on that session, to learn more about it.

P2, male, IG

In addition, becoming aware of good sleep practices, such as following a night-time sleep routine (plan), may have motivated participants to “stick” to their behavioral changes: “I found the information mostly useful about the sleep plan—it helped, because I just stuck to the plan afterwards” [P9, female, IG]. Maintaining behavioral changes may have been encouraged by noticing improvements in sleep and health, such as feeling refreshed upon waking: “I actually felt a lot better—especially being off my phone, I felt more refreshed in the morning when I woke up” [P13, female, IG], or improved night-time sleep: “I don’t actually wake up in the night anymore—I can actually sleep through, which is good” [P7, female, IG].

## Discussion

### Principal Findings

SleepWise shows promise as a potentially efficacious intervention for improving sleep quality among adolescents, as it was engaged with and deemed acceptable across both trials. The first session of the intervention (eating and sleep) was highly favored by adolescents, along with session 4 (understanding how everyday habits and environments impact sleep). Incentivization did not greatly influence intervention engagement, acceptability, or sleep quality, with participants in the nonincentivized group showing more success in achieving their set goals (ie, reported “meeting” their set goals more frequently). It was clear from participant feedback that adolescents lead busy lifestyles; however, the intervention delivery format was deemed time-efficient and suited participants’ busy schedules, albeit some further refinements to improve flexibility and personalization could be beneficial. Encouragingly, adolescents monitored their sleep behaviors using the sleep diary and goal-setting features of the intervention and attempted valuable changes to sleep-related behaviors (eg, reducing screen time before sleep).

### Comparison With Previous Research

The findings from this study suggest that incentivization does not greatly influence the acceptability of the intervention or sleep quality, with the nonincentivized trial (trial 2) showing a larger effect size, suggesting that participants in the nonincentivized trial showed a greater improvement in sleep quality. However, those in the incentivized trial viewed slightly more pages than those in the nonincentivized trial, suggesting that participants in the incentivized trial may have been more motivated to work through the intervention systematically. While this finding may be explained by individuals’ inclination to be more motivated by instant and guaranteed, versus delayed and lottery-based, incentives [44,45], those in the nonincentivized trial achieved their set goals more frequently, suggesting that incentivization may not necessarily encourage higher levels of behavior change. Clearly, using incentives in research requires careful consideration. The effectiveness and maintenance of behavior change when using incentives, especially when targeted at more challenging and complex behaviors and at different populations, is not well understood, and their feasibility is perhaps even less so [46-49]. While evidence suggests that health-promoting incentives have the potential to promote behavior change [50], controversy exists over the form and level of incentives, as there may be an expectation that incentives could be more attractive and effective for certain individuals, and therefore risk stigmatization or coercion [51,52]. Adding to this complexity, younger people may have a more favorable view toward incentives [49], yet the provision of incentives to adolescents remains unclear; therefore, careful consideration should be taken [53]. Nevertheless, the use of incentives may only be feasible to the extent to which they are deemed cost-effective to implement in practice when provided alongside health education and behavioral support [51,54]. Therefore, as the findings from this study suggest, incentivizing sleep interventions for adolescents may not impact acceptability, sleep quality and engagement with web-based sleep interventions, which may prove cost-effective in the context of a definitive trial.

Adolescents in this study viewed the intervention more favorably when the session content was deemed relevant to their needs (eg, age-appropriate), as well as having flexibility and easily accessible and personalized intervention features. In line with these findings, Werner-Seidler et al [55] show that adolescents deem an app-based sleep intervention more acceptable if the intervention content is tailored to their age group, its use is flexible, and the intervention can be personalized to each user. Personalized and flexible approaches to intervention delivery may also have the added benefit of reflecting users’ specific needs and allowing adaptive and targeted communication. With adolescents strongly favoring personalized and flexible approaches to intervention delivery, more innovative approaches to developing sleep interventions could replace one-off activity monitors and sleep logs [13], which may in turn decrease clinical time and increase availability and accessibility to treatment options for adolescents with sleep-related problems [13,56].

The findings from this study highlight the importance for sleep interventions to align their design goals with adolescents’ priorities and values. Adolescents in this study expressed leading busy lifestyles (eg, completing high volumes of homework), which may hinder their engagement with the intervention. As may be expected, high levels of homework [22], coupled with other adolescent-specific priorities such as maintaining social relationships [22,57], may make it more difficult to engage with sleep interventions. Given that adolescents face a unique set of age-related barriers, such as time constraints [58], and attending to early school start times [59], enabling bite-sized (eg, short weekly sessions) and interactive digital communications via platforms such as SleepWise could encourage adolescents to engage with interventions and subsequent sleep behavior change. Indeed, adolescents in this study reported attempts at behavior change when the intervention content was deemed important and relevant to them, suggesting that participants’ intention to change behavior may be influenced by how acceptable they deem an intervention (eg, interesting and time-efficient). With research indicating that adolescents prefer time-efficient and age-appropriate interventions [20,55], and adolescence being a potential marker for reduced application of intentional health-related self-regulatory behaviors [60], and characterized by increased impulsivity [61], it becomes critical to align interventions such as SleepWise with adolescents’ priorities and take into account the contextual factors (eg, academic commitments) that may facilitate or hinder engagement and subsequent behavior change [62].

### Limitations

Limitations of this study include the lack of an active CG. While adolescents in the CG continued to receive their usual education, it may have been preferential to include an active CG or psychological placebo. Additionally, a cluster-randomized trial was not undertaken. Implementing a cluster-randomized trial would reduce the likelihood of cross-contamination.

This study had several strengths. First, a novel approach that puts the voices of adolescent users at the center of intervention development was undertaken, ensuring the intervention was in line with participants’ priorities. Second, SleepWise was evaluated in the context of a feasibility trial, which helped answer key feasibility questions (eg, acceptability of the intervention) and contributed to reducing research waste and time. Lastly, and regarding the qualitative methodology used in this study, a strong commitment was made to prolonged engagement with the topic and undertaking a rigorous approach to qualitative data collection and analysis. Rigor in this sense refers to the elements of data that were collected and analyzed in relation to supplying all the information required for a comprehensive qualitative analysis [63].

### Conclusions

This study addresses a core gap in the literature by testing the feasibility and potential efficacy of a person-based and web-based sleep intervention (SleepWise) aimed at adolescents. The SleepWise study has laid the foundation for developing an understanding of adolescents’ engagement and acceptability with web-based sleep interventions, with adolescents in this study perceiving SleepWise as an acceptable intervention (eg, time-efficient) with recommendations for future improvements (eg, personalizing the goal-setting feature). Interestingly, incentivization of the intervention did not show a notably large impact on engagement, acceptability, or sleep quality. Effect size analysis showed promise for improved sleep quality across both trials; thus, a main trial is warranted to test the effectiveness of SleepWise.

## Appendix

Multimedia Appendix 1 SleepWise quiz example.

Multimedia Appendix 2 SleepWise educational game example.

Multimedia Appendix 3 CONSORT-eHEALTH checklist (V 1.6.1).

## Abbreviations

CBT: cognitive behavioral therapy
CBT-I: cognitive behavioral therapy for insomnia
CG: control group
IG: intervention group
PBA: person-based approach
PSQI-SF: Pittsburgh Sleep Quality Index–Short Form

## Data Availability

The data sets generated and analyzed during this study are available from the corresponding author on reasonable request.
