# Supplementary figures and images for: A Person-Based Web-Based Sleep Intervention Aimed at Adolescents (SleepWise): Randomized Controlled Feasibility Study
Source: JMIR Form Res. 2024 Oct 23;8:e51322. doi: 10.2196/51322 (PMC11541153; doi:10.2196/51322)

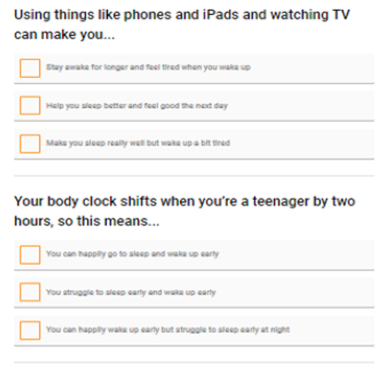

Supplement: Multimedia Appendix 1 [file formative_v8i1e51322_app1.png]

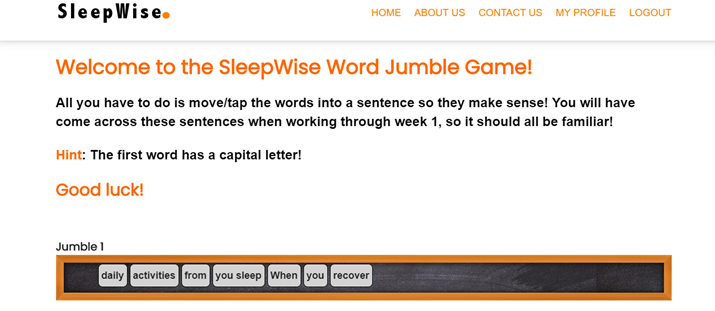

Supplement: Multimedia Appendix 2 [file formative_v8i1e51322_app2.png]
