# Supplementary material for: A Person-Based Web-Based Sleep Intervention Aimed at Adolescents (SleepWise): Randomized Controlled Feasibility Study
Source: JMIR Form Res. 2024 Oct 23;8:e51322. doi: 10.2196/51322 (PMC11541153; doi:10.2196/51322)
Supplement: Multimedia Appendix 3 [file formative_v8i1e51322_app3.pdf]

CONSORT-EHEALTH (V 1.6.1) - Submission/Publication Form

The CONSORT-EHEALTH checklist is intended for authors of randomized trials evaluating web-based and Internet-based applications/interventions, including mobile interventions, electronic games (incl multiplayer games), social media, certain telehealth applications, and other interactive and/or networked electronic applications. Some of the items (e.g. all subitems under item 5 - description of the intervention) may also be applicable for other study designs.

The goal of the CONSORT EHEALTH checklist and guideline is to be
a) a guide for reporting for authors of RCTs,
b) to form a basis for appraisal of an ehealth trial (in terms of validity)

CONSORT-EHEALTH items/subitems are MANDATORY reporting items for studies published in the Journal of Medical Internet Research and other journals / scientific societies endorsing the checklist.

Items numbered 1., 2., 3., 4a., 4b etc are original CONSORT or CONSORT-NPT (non-pharmacologic treatment) items.
Items with Roman numerals (i., ii, iii, iv etc.) are CONSORT-EHEALTH extensions/clarifications.

As the CONSORT-EHEALTH checklist is still considered in a formative stage, we would ask that you also RATE ON A SCALE OF 1-5 how important/useful you feel each item is FOR THE PURPOSE OF THE CHECKLIST and reporting guideline (optional).

Mandatory reporting items are marked with a red *.
In the textboxes, either copy & paste the relevant sections from your manuscript into this

Your response is too large. Try shortening some answers.

on why the item was not relevant for this study.

YOUR ANSWERS WILL BE PUBLISHED AS A SUPPLEMENTARY FILE TO YOUR PUBLICATION IN JMIR AND ARE CONSIDERED PART OF YOUR PUBLICATION (IF ACCEPTED).
Please fill in these questions diligently. Information will not be copyedited, so please use proper spelling and grammar, use correct capitalization, and avoid abbreviations.

DO NOT FORGET TO SAVE AS PDF _AND_ CLICK THE SUBMIT BUTTON SO YOUR ANSWERS ARE IN OUR DATABASE !!!

Citation Suggestion (if you append the pdf as Appendix we suggest to cite this paper in the caption):
Eysenbach G, CONSORT-EHEALTH Group
CONSORT-EHEALTH: Improving and Standardizing Evaluation Reports of Web-based and Mobile Health Interventions
J Med Internet Res 2011;13(4):e126
URL: http://www.jmir.org/2011/4/e126/
doi: 10.2196/jmir.1923
PMID: 22209829

shokranehmoghadam@yahoo.co.uk (not shared)
Switch account

Draft not saved

* Required

Your name *
First Last

Shokraneh Moghadam

Your response is too large. Try shortening some answers.

Primary Affiliation (short), City, Country *

University of Toronto, Toronto, Canada

University of Exeter

Your e-mail address *

abc@gmail.com

s.oftadeh-moghadam@exeter.ac.uk

Title of your manuscript *

Provide the (draft) title of your manuscript.

The SleepWise Study: A randomised controlled feasibility trial of a person-based web-based sleep intervention aimed at adolescents

Your response is too large. Try shortening some answers.

Name of your App/Software/Intervention *

If there is a short and a long/alternate name, write the short name first and add the long name in brackets.

SleepWise

Evaluated Version (if any)

e.g. "V1", "Release 2017-03-01", "Version 2.0.27913"

Your answer

Language(s) *

What language is the intervention/app in? If multiple languages are available, separate by comma (e.g. "English, French")

English

Your response is too large. Try shortening some answers.

URL of your Intervention Website or App

e.g. a direct link to the mobile app on app in appstore (itunes, Google Play), or URL of the website. If the intervention is a DVD or hardware, you can also link to an Amazon page.

Your answer

URL of an image/screenshot (optional)

Your answer

Accessibility *

Can an enduser access the intervention presently?

- ○ access is free and open
- ○ access only for special usergroups, not open
- ○ access is open to everyone, but requires payment/subscription/in-app purchases
- ○ app/intervention no longer accessible
- ◉ Other: The website is currently inactive and therefore cannot be accessed.

Your response is too large. Try shortening some answers.

Primary Medical Indication/Disease/Condition *

e.g. "Stress", "Diabetes", or define the target group in brackets after the condition, e.g. "Autism (Parents of children with)", "Alzheimers (Informal Caregivers of)"

Sleep

Primary Outcomes measured in trial *

comma-separated list of primary outcomes reported in the trial

Sleep Quality, Acceptability

Secondary/other outcomes

Are there any other outcomes the intervention is expected to affect?

Your answer

Your response is too large. Try shortening some answers.

Recommended "Dose" *

What do the instructions for users say on how often the app should be used?

- ( ) Approximately Daily
- (•) Approximately Weekly
- ( ) Approximately Monthly
- ( ) Approximately Yearly
- ( ) "as needed"
- ( ) Other:

Your response is too large. Try shortening some answers.

Approx. Percentage of Users (starters) still using the app as recommended after 3 months *

- (selected) unknown / not evaluated
- 0-10%
- 11-20%
- 21-30%
- 31-40%
- 41-50%
- 51-60%
- 61-70%
- 71%-80%
- 81-90%
- 91-100%
- Other:

Your response is too large. Try shortening some answers.

Overall, was the app/intervention effective? *

- ○ yes: all primary outcomes were significantly better in intervention group vs control
- ○ partly: SOME primary outcomes were significantly better in intervention group vs control
- ○ no statistically significant difference between control and intervention
- ○ potentially harmful: control was significantly better than intervention in one or more outcomes
- ○ inconclusive: more research is needed
- ◉ Other: The intervention was tested as part of a feasibility study and showed [illegible]

Your response is too large. Try shortening some answers.

Article Preparation Status/Stage *

At which stage in your article preparation are you currently (at the time you fill in this form)

- ( ) not submitted yet - in early draft status
- (x) not submitted yet - in late draft status, just before submission
- ( ) submitted to a journal but not reviewed yet
- ( ) submitted to a journal and after receiving initial reviewer comments
- ( ) submitted to a journal and accepted, but not published yet
- ( ) published
- ( ) Other:

Your response is too large. Try shortening some answers.

Journal *

If you already know where you will submit this paper (or if it is already submitted), please provide the journal name (if it is not JMIR, provide the journal name under "other")

- ( ) not submitted yet / unclear where I will submit this
- ( ) Journal of Medical Internet Research (JMIR)
- ( ) JMIR mHealth and UHealth
- ( ) JMIR Serious Games
- ( ) JMIR Mental Health
- ( ) JMIR Public Health
- (x) JMIR Formative Research
- ( ) Other JMIR sister journal
- ( ) Other:

Is this a full powered effectiveness trial or a pilot/feasibility trial? *

- (x) Pilot/feasibility
- ( ) Fully powered

Your response is too large. Try shortening some answers.

Manuscript tracking number *

If this is a JMIR submission, please provide the manuscript tracking number under "other" (The ms tracking number can be found in the submission acknowledgement email, or when you login as author in JMIR. If the paper is already published in JMIR, then the ms tracking number is the four-digit number at the end of the DOI, to be found at the bottom of each published article in JMIR)

- (selected) no ms number (yet) / not (yet) submitted to / published in JMIR
- Other:

TITLE AND ABSTRACT

1a) TITLE: Identification as a randomized trial in the title

1a) Does your paper address CONSORT item 1a? *

I.e does the title contain the phrase "Randomized Controlled Trial"? (if not, explain the reason under "other")

- (selected) yes
- Other:

Your response is too large. Try shortening some answers.

1a-i) Identify the mode of delivery in the title

Identify the mode of delivery. Preferably use “web-based” and/or “mobile” and/or “electronic game” in the title. Avoid ambiguous terms like “online”, “virtual”, “interactive”. Use “Internet-based” only if Intervention includes non-web-based Internet components (e.g. email), use “computer-based” or “electronic” only if offline products are used. Use “virtual” only in the context of “virtual reality” (3-D worlds). Use “online” only in the context of “online support groups”. Complement or substitute product names with broader terms for the class of products (such as “mobile” or “smart phone” instead of “iphone”), especially if the application runs on different platforms.

	1	2	3	4	5	
subitem not at all important	○	○	○	○	◉	essential

Clear selection

Does your paper address subitem 1a-i? *

Copy and paste relevant sections from manuscript title (include quotes in quotation marks "like this" to indicate direct quotes from your manuscript), or elaborate on this item by providing additional information not in the ms, or briefly explain why the item is not applicable/relevant for your study

Yes - "The SleepWise Study: A randomised controlled feasibility trial of a person-based web-based sleep intervention aimed at adolescents"

Your response is too large. Try shortening some answers.

1a-ii) Non-web-based components or important co-interventions in title

Mention non-web-based components or important co-interventions in title, if any (e.g., “with telephone support”).

	1	2	3	4	5	
subitem not at all important	◉	○	○	○	○	essential

Clear selection

Does your paper address subitem 1a-ii?

Copy and paste relevant sections from manuscript title (include quotes in quotation marks "like this" to indicate direct quotes from your manuscript), or elaborate on this item by providing additional information not in the ms, or briefly explain why the item is not applicable/relevant for your study

This item is not applicable/relevant to this paper as the intervention did not include non-web-based components or important co-interventions.

Your response is too large. Try shortening some answers.

1a-iii) Primary condition or target group in the title

Mention primary condition or target group in the title, if any (e.g., “for children with Type I Diabetes”) Example: A Web-based and Mobile Intervention with Telephone Support for Children with Type I Diabetes: Randomized Controlled Trial

	1	2	3	4	5	
subitem not at all important	○	○	○	○	◉	essential

Clear selection

Does your paper address subitem 1a-iii? *

Copy and paste relevant sections from manuscript title (include quotes in quotation marks "like this" to indicate direct quotes from your manuscript), or elaborate on this item by providing additional information not in the ms, or briefly explain why the item is not applicable/relevant for your study

Yes - "The SleepWise Study: A randomised controlled feasibility trial of a person-based web-based sleep intervention aimed at adolescents"

1b) ABSTRACT: Structured summary of trial design, methods, results, and conclusions

NPT extension: Description of experimental treatment, comparator, care providers, centers, [illegible]

Your response is too large. Try shortening some answers.

1b-i) Key features/functionalities/components of the intervention and comparator in the METHODS section of the ABSTRACT

Mention key features/functionalities/components of the intervention and comparator in the abstract. If possible, also mention theories and principles used for designing the site. Keep in mind the needs of systematic reviewers and indexers by including important synonyms. (Note: Only report in the abstract what the main paper is reporting. If this information is missing from the main body of text, consider adding it)

	1	2	3	4	5	
subitem not at all important	○	○	○	◉	○	essential

Clear selection

Does your paper address subitem 1b-i? *

Copy and paste relevant sections from the manuscript abstract (include quotes in quotation marks "like this" to indicate direct quotes from your manuscript), or elaborate on this item by providing additional information not in the ms, or briefly explain why the item is not applicable/relevant for your study

Yes - "A feasibility trial was conducted to test the feasibility and acceptability of a web-based sleep intervention, called SleepWise, developed based on the Person-Based Approach to intervention development. Ninety participants (aged 13 to 17) from Further Education institutions and secondary schools were recruited to two, 2-arm randomized controlled trials. One trial (Trial 1) included incentives to understand the impact of participant incentives on engagement with the intervention.".

Your response is too large. Try shortening some answers.

1b-ii) Level of human involvement in the METHODS section of the ABSTRACT

Clarify the level of human involvement in the abstract, e.g., use phrases like “fully automated” vs. “therapist/nurse/care provider/physician-assisted” (mention number and expertise of providers involved, if any). (Note: Only report in the abstract what the main paper is reporting. If this information is missing from the main body of text, consider adding it)

	1	2	3	4	5	
subitem not at all important	○	○	◉	○	○	essential

Clear selection

Does your paper address subitem 1b-ii?

Copy and paste relevant sections from the manuscript abstract (include quotes in quotation marks "like this" to indicate direct quotes from your manuscript), or elaborate on this item by providing additional information not in the ms, or briefly explain why the item is not applicable/relevant for your study

This item is not applicable/relevant to this paper as no level of human contact was provided as part of the intervention i.e., there were no human support/assisted components to the intervention.

Your response is too large. Try shortening some answers.

1b-iii) Open vs. closed, web-based (self-assessment) vs. face-to-face assessments in the METHODS section of the ABSTRACT

Mention how participants were recruited (online vs. offline), e.g., from an open access website or from a clinic or a closed online user group (closed usergroup trial), and clarify if this was a purely web-based trial, or there were face-to-face components (as part of the intervention or for assessment). Clearly say if outcomes were self-assessed through questionnaires (as common in web-based trials). Note: In traditional offline trials, an open trial (open-label trial) is a type of clinical trial in which both the researchers and participants know which treatment is being administered. To avoid confusion, use “blinded” or “unblinded” to indicated the level of blinding instead of “open”, as “open” in web-based trials usually refers to “open access” (i.e. participants can self-enrol). (Note: Only report in the abstract what the main paper is reporting. If this information is missing from the main body of text, consider adding it)

	1	2	3	4	5	
subitem not at all important	○	○	◉	○	○	essential

Clear selection

Your response is too large. Try shortening some answers.

Does your paper address subitem 1b-iii?

Copy and paste relevant sections from the manuscript abstract (include quotes in quotation marks "like this" to indicate direct quotes from your manuscript), or elaborate on this item by providing additional information not in the ms, or briefly explain why the item is not applicable/relevant for your study

This item is not applicable/relevant to this paper as the study did not include any face to face components (the intervention was wholly web-based), there were no components of blinding, and further details on how questionnaire data was collected is detailed in the main body of the paper under section titled "Measures".

1b-iv) RESULTS section in abstract must contain use data

Report number of participants enrolled/assessed in each group, the use/uptake of the intervention (e.g., attrition/adherence metrics, use over time, number of logins etc.), in addition to primary/secondary outcomes. (Note: Only report in the abstract what the main paper is reporting. If this information is missing from the main body of text, consider adding it)

	1	2	3	4	5	
subitem not at all important	○	○	○	◉	○	essential

Clear selection

Your response is too large. Try shortening some answers.

Does your paper address subitem 1b-iv?

Copy and paste relevant sections from the manuscript abstract (include quotes in quotation marks "like this" to indicate direct quotes from your manuscript), or elaborate on this item by providing additional information not in the ms, or briefly explain why the item is not applicable/relevant for your study

Yes - "Participants reported high levels of acceptability across both trials (Trial 1 M 21.00 (2.74); Trial 2 M 20.82 (2.48) and found SleepWise to be an engaging and suitable sleep programme for their age group. Both trials demonstrated a significant improvement in sleep quality scores for the intervention, as opposed to control groups, with a medium (Trial 1) and large (Trial 2) effect size. A larger effect size for improvement in sleep quality was found in the non-incentivized trial (d = .87), suggesting that incentivization may not impact engagement and/or sleep quality outcomes. Overall, findings suggest that SleepWise is potentially efficacious and could feasibly be taken forward into a definitive main trial."

1b-v) CONCLUSIONS/DISCUSSION in abstract for negative trials

Conclusions/Discussions in abstract for negative trials: Discuss the primary outcome - if the trial is negative (primary outcome not changed), and the intervention was not used, discuss whether negative results are attributable to lack of uptake and discuss reasons. (Note: Only report in the abstract what the main paper is reporting. If this information is missing from the main body of text, consider adding it)

	1	2	3	4	5	
subitem not at all important	○	◉	○	○	○	essential

Your response is too large. Try shortening some answers.

Does your paper address subitem 1b-v?

Copy and paste relevant sections from the manuscript abstract (include quotes in quotation marks "like this" to indicate direct quotes from your manuscript), or elaborate on this item by providing additional information not in the ms, or briefly explain why the item is not applicable/relevant for your study

This item is not wholly applicable/relevant to this paper as the study was a feasibility study and therefore the primary outcome was not tested for effectiveness however preliminary indicators of efficaciousness and intervention acceptability are mentioned in the conclusion: "SleepWise is a pragmatic solution to an important public health issue, and an acceptable and potentially efficacious web-based intervention to improve adolescent sleep"

INTRODUCTION

2a) In INTRODUCTION: Scientific background and explanation of rationale

Your response is too large. Try shortening some answers.

2a-i) Problem and the type of system/solution

Describe the problem and the type of system/solution that is object of the study: intended as stand-alone intervention vs. incorporated in broader health care program? Intended for a particular patient population? Goals of the intervention, e.g., being more cost-effective to other interventions, replace or complement other solutions? (Note: Details about the intervention are provided in “Methods” under 5)

	1	2	3	4	5	
subitem not at all important	○	○	○	○	◉	essential

Clear selection

Your response is too large. Try shortening some answers.

Does your paper address subitem 2a-i? *

Copy and paste relevant sections from the manuscript (include quotes in quotation marks "like this" to indicate direct quotes from your manuscript), or elaborate on this item by providing additional information not in the ms, or briefly explain why the item is not applicable/relevant for your study

Yes - "Poor sleep quality among adolescents is an important public health problem. Research show that 53% of adolescents get less than 8-hours of sleep per night, and do not sleep for the recommended amount of 8 to 10 hours, especially on school night...there are several interventions aimed at improving sleep quality in adolescents. These include school-based sleep education programmes, which have been shown to successfully improve sleep knowledge in adolescents, but to be less useful for improving sleep behavior...Interventions aimed at targeting specific behaviors, such as reducing screen time before sleep, have proven somewhat more successful for improving sleep problems and sleep behaviors among adolescents. Likewise, interventions that include cognitive behavior therapy (CBT) have been found to be successful, producing significant improvements in both objective and subjective sleep outcomes among adolescent...Determining the success of sleep interventions is difficult, as research in this area is often limited by a lack of control groups and the application of a strong theoretical foundation, which makes it difficult to determine the mechanisms that make an intervention effective. Moreover, these interventions are frequently viewed by participants as undesirable as they are often non-interactive and time-consuming. Coupled with these shortcomings, sleep interventions targeted at adolescents are also limited by small sample sizes, high dropout rates, little to no follow-up data, and are typically costly...One clear recommendation is for intervention developers to use digital platforms, which offer an attractive, feasible, and effective method to health management, including sleep behaviors among adolescents...This study aims to overcome the limitations of previous adolescent sleep interventions, by undertaking a feasibility trial to investigate the acceptability and feasibility of a theory-evidence and person-based web-based sleep intervention called SleepWise. The aim of this feasibility study is to examine the acceptability, feasibility, and preliminary effects of SleepWise."

Your response is too large. Try shortening some answers.

2a-ii) Scientific background, rationale: What is known about the (type of) system

Scientific background, rationale: What is known about the (type of) system that is the object of the study (be sure to discuss the use of similar systems for other conditions/diagnoses, if appropiate), motivation for the study, i.e. what are the reasons for and what is the context for this specific study, from which stakeholder viewpoint is the study performed, potential impact of findings [2]. Briefly justify the choice of the comparator.

	1	2	3	4	5	
subitem not at all important	○	○	○	●	○	essential

Clear selection

Your response is too large. Try shortening some answers.

Does your paper address subitem 2a-ii? *

Copy and paste relevant sections from the manuscript (include quotes in quotation marks "like this" to indicate direct quotes from your manuscript), or elaborate on this item by providing additional information not in the ms, or briefly explain why the item is not applicable/relevant for your study

Please see the next page for answer.

Your response is too large. Try shortening some answers.

Yes - "Poor sleep quality among adolescents is an important public health problem1–3. Research show that 53% of adolescents get less than 8-hours of sleep per night, and do not sleep for the recommended amount of 8 to 10 hours, especially on school nights. Additionally, at least 36% of adolescents experience difficulty falling asleep, with 59% waking up feeling tired during the week 4–8. Poor sleep quality in adolescents is associated with detrimental health outcomes, including unhealthy weight gain, reduced levels of physical activity, increased risk-taking behaviors, and increased levels of depression and anxiety, which all risk continuation into adulthood...there are several interventions aimed at improving sleep quality in adolescents. These include school-based sleep education programmes, which have been shown to successfully improve sleep knowledge in adolescents, but to be less useful for improving sleep behavior...Interventions aimed at targeting specific behaviors, such as reducing screen time before sleep, have proven somewhat more successful for improving sleep problems and sleep behaviors among adolescents. Likewise, interventions that include cognitive behavior therapy (CBT) have been found to be successful, producing significant improvements in both objective and subjective sleep outcomes among adolescent...Determining the success of sleep interventions is difficult, as research in this area is often limited by a lack of control groups and the application of a strong theoretical foundation, which makes it difficult to determine the mechanisms that make an intervention effective. Moreover, these interventions are frequently viewed by participants as undesirable as they are often non-interactive and time-consuming. Coupled with these shortcomings, sleep interventions targeted at adolescents are also limited by small sample sizes, high dropout rates, little to no follow-up data, and are typically costly...There is an urgent need for innovative solutions in this field of research. One clear recommendation is for intervention developers to use digital platforms, which offer an attractive, feasible, and effective method to health management, including sleep behaviors among adolescents...There has already been some success in this field. For example, meta-analyses show that the effects of digitalized CBT programmes for insomnia (eCBT-I) are similar to those for face-to-face, indicating improvements in insomnia severity, sleep efficiency, subjective sleep quality, wake after sleep onset, sleep onset latency, total sleep time, and number of nocturnal awakenings. These effects were comparable to those found with face-to-face CBT-I programmes and were commonly maintained at 4-48 weeks follow-up.". Please see manuscript for further details.

2b) In INTRODUCTION: Specific objectives or hypotheses

Does your paper address CONSORT subitem 2b? *

Copy and paste relevant sections from the manuscript (include quotes in quotation marks "like this" to indicate direct quotes from your manuscript), or elaborate on this item by providing additional information not in the ms, or briefly explain why the item is not applicable/relevant for your study

Yes - "This study aims to overcome the limitations of previous adolescent sleep interventions, by undertaking a feasibility trial to investigate the acceptability and feasibility of a theory-evidence and person-based web-based sleep intervention called SleepWise. The aim of this feasibility study is to examine the acceptability, feasibility, and preliminary effects of SleepWise. In line with guidelines on the development of complex interventions, the primary purpose of this study is to explore recruitment rates, attrition, and completion, and to conduct a mixed methods process evaluation to determine the acceptability of SleepWise among adolescents. Two separate randomized controlled feasibility trials were undertaken as part of this study; Trial 1 was incentivized, and Trial 2 was not. This was done to better understand the impact of incentivization on adolescent's engagement with the intervention, as the use of incentives need careful consideration and understanding in research. A final aim is to investigate the preliminary impact of SleepWise on sleep quality.".

METHODS

Your response is too large. Try shortening some answers.

3a) Description of trial design (such as parallel, factorial) including allocation ratio

Does your paper address CONSORT subitem 3a? *

Copy and paste relevant sections from the manuscript (include quotes in quotation marks "like this" to indicate direct quotes from your manuscript), or elaborate on this item by providing additional information not in the ms, or briefly explain why the item is not applicable/relevant for your study

Yes - description of the trial design is included in the methods section of this paper: "Two separate randomized controlled feasibility trials were undertaken as part of this study. The only difference between the trials was that Trial 1 was incentivized and Trial 2 was not. This was to determine the impact of incentivization as part of the feasibility study. Trial 1 recruitment took place from November 2018 to May 2019 (study running in the summer term), with a rolling start time. Trial 2 recruitment took place from May 2019 to December 2019 (study running in the autumn term), also with a rolling start time. Ethical approval for the study was received from the University's ethics committee
(XXX), and the study was undertaken in accordance with the Helsinki Declaration"

3b) Important changes to methods after trial commencement (such as eligibility criteria), with reasons

Your response is too large. Try shortening some answers.

Does your paper address CONSORT subitem 3b? *

Copy and paste relevant sections from the manuscript (include quotes in quotation marks "like this" to indicate direct quotes from your manuscript), or elaborate on this item by providing additional information not in the ms, or briefly explain why the item is not applicable/relevant for your study

This items is not relevant/applicable to this paper as no important changes were made to the methods after trial commencement.

3b-i) Bug fixes, Downtimes, Content Changes

Bug fixes, Downtimes, Content Changes: ehealth systems are often dynamic systems. A description of changes to methods therefore also includes important changes made on the intervention or comparator during the trial (e.g., major bug fixes or changes in the functionality or content) (5-iii) and other “unexpected events” that may have influenced study design such as staff changes, system failures/downtimes, etc. [2].

	1	2	3	4	5	
subitem not at all important	◉	○	○	○	○	essential

Clear selection

Your response is too large. Try shortening some answers.

Does your paper address subitem 3b-i?

Copy and paste relevant sections from the manuscript (include quotes in quotation marks "like this" to indicate direct quotes from your manuscript), or elaborate on this item by providing additional information not in the ms, or briefly explain why the item is not applicable/relevant for your study

This items is not relevant/applicable to this paper as no important changes were made to the intervention during the trial.

4a) Eligibility criteria for participants

Does your paper address CONSORT subitem 4a? *

Copy and paste relevant sections from the manuscript (include quotes in quotation marks "like this" to indicate direct quotes from your manuscript), or elaborate on this item by providing additional information not in the ms, or briefly explain why the item is not applicable/relevant for your study

Yes - "The eligibility criteria for all parts of the study, included being aged between 13 to 19 years and having access to a computer/laptop with internet access. Participants were not screened for any sleep, mental, or physical health conditions, as SleepWise is intended for use in a general population of adolescents, and not in an exclusive clinical or sub-clinical group.".

Your response is too large. Try shortening some answers.

4a-i) Computer / Internet literacy

Computer / Internet literacy is often an implicit “de facto” eligibility criterion - this should be explicitly clarified.

	1	2	3	4	5	
subitem not at all important	○	◉	○	○	○	essential

Clear selection

Does your paper address subitem 4a-i?

Copy and paste relevant sections from the manuscript (include quotes in quotation marks "like this" to indicate direct quotes from your manuscript), or elaborate on this item by providing additional information not in the ms, or briefly explain why the item is not applicable/relevant for your study

This item is no wholly relevant/applicable to this paper as it was assumed that participants who consented to take part in this study had met the eligibility criteria of "having access to a computer/laptop with internet access" and were therefore literate in its use.

Your response is too large. Try shortening some answers.

4a-ii) Open vs. closed, web-based vs. face-to-face assessments:

Open vs. closed, web-based vs. face-to-face assessments: Mention how participants were recruited (online vs. offline), e.g., from an open access website or from a clinic, and clarify if this was a purely web-based trial, or there were face-to-face components (as part of the intervention or for assessment), i.e., to what degree got the study team to know the participant. In online-only trials, clarify if participants were quasi-anonymous and whether having multiple identities was possible or whether technical or logistical measures (e.g., cookies, email confirmation, phone calls) were used to detect/prevent these.

	1	2	3	4	5	
subitem not at all important	○	○	○	◉	○	essential

Clear selection

Your response is too large. Try shortening some answers.

Does your paper address subitem 4a-ii? *

Copy and paste relevant sections from the manuscript (include quotes in quotation marks "like this" to indicate direct quotes from your manuscript), or elaborate on this item by providing additional information not in the ms, or briefly explain why the item is not applicable/relevant for your study

Yes - "Participants were recruited from three secondary schools and two Further Education colleges in South East England...Participant recruitment happened via the researcher reaching out to schools/colleges advertising the study and sending posters to those who responded as interested. An allocated member of staff (study facilitator) at each study site (schools/ colleges) then sent out a poster advertising the study to their students. Sites were offered an assembly or short talk advertising the study; if they chose this option, the researcher played a short video about adolescent health and talked about the aims of the study. Adolescents who were interested in partaking in the study collected consent and information sheets from the study facilitator (ie., site staff). Signed consent forms were then collected by the researcher from each site. Parental consent was not required for participants aged 16 years and over, as per the British Psychological Society's (BPS) ethical guidelines.".

Your response is too large. Try shortening some answers.

4a-iii) Information giving during recruitment

Information given during recruitment. Specify how participants were briefed for recruitment and in the informed consent procedures (e.g., publish the informed consent documentation as appendix, see also item X26), as this information may have an effect on user self-selection, user expectation and may also bias results.

	1	2	3	4	5	
subitem not at all important	○	○	○	○	◉	essential

Clear selection

Your response is too large. Try shortening some answers.

Does your paper address subitem 4a-iii?

Copy and paste relevant sections from the manuscript (include quotes in quotation marks "like this" to indicate direct quotes from your manuscript), or elaborate on this item by providing additional information not in the ms, or briefly explain why the item is not applicable/relevant for your study

Yes - "Participants were recruited from three secondary schools and two Further Education colleges in South East England...Participant recruitment happened via the researcher reaching out to schools advertising the study and sending posters to those who responded as interested. An allocated member of staff (study facilitator) at each study site (schools) then sent out a poster advertising the study to their students. Sites were offered an assembly or short talk advertising the study; if they chose this option, the researcher played a short video about sleep and health and talked about the aims of the study. Adolescents who were interested in partaking in the study collected consent and information sheets from the study facilitator (ie., site staff). Signed consent forms were then collected by the researcher from each site. Parental consent was not required for participants aged 16 years and over, as per the British Psychological Society's (BPS) ethical guidelines."

4b) Settings and locations where the data were collected

Your response is too large. Try shortening some answers.

Does your paper address CONSORT subitem 4b? *

Copy and paste relevant sections from the manuscript (include quotes in quotation marks "like this" to indicate direct quotes from your manuscript), or elaborate on this item by providing additional information not in the ms, or briefly explain why the item is not applicable/relevant for your study

Yes - "Participants were recruited from three secondary schools and two Further Education colleges in South East England...Participant recruitment happened via the researcher reaching out to schools advertising the study and sending posters to those who responded as interested. An allocated member of staff (study facilitator) at each study site (schools) then sent out a poster advertising the study to their students. Sites were offered an assembly or short talk advertising the study; if they chose this option, the researcher played a short video about sleep and health and talked about the aims of the study. Adolescents who were interested in partaking in the study collected consent and information sheets from the study facilitator (ie., site staff). Signed consent forms were then collected by the researcher from each site. Parental consent was not required for participants aged 16 years and over, as per the British Psychological Society's (BPS) ethical guidelines".

4b-i) Report if outcomes were (self-)assessed through online questionnaires

Clearly report if outcomes were (self-)assessed through online questionnaires (as common in web-based trials) or otherwise.

	1	2	3	4	5	
subitem not at all important	○	○	○	○	◉	essential

Your response is too large. Try shortening some answers.

Does your paper address subitem 4b-i? *

Copy and paste relevant sections from the manuscript (include quotes in quotation marks "like this" to indicate direct quotes from your manuscript), or elaborate on this item by providing additional information not in the ms, or briefly explain why the item is not applicable/relevant for your study

Yes- "...participants completed a baseline measure questionnaire about their sleep quality via Qualtrics (an online data administration software)..."

4b-ii) Report how institutional affiliations are displayed

Report how institutional affiliations are displayed to potential participants [on ehealth media], as affiliations with prestigious hospitals or universities may affect volunteer rates, use, and reactions with regards to an intervention.(Not a required item – describe only if this may bias results)

	1	2	3	4	5	
subitem not at all important	○	○	◉	○	○	essential

Clear selection

Your response is too large. Try shortening some answers.

Does your paper address subitem 4b-ii?

Copy and paste relevant sections from the manuscript (include quotes in quotation marks "like this" to indicate direct quotes from your manuscript), or elaborate on this item by providing additional information not in the ms, or briefly explain why the item is not applicable/relevant for your study

This item is not wholly relevant/applicable to this paper as the institution was not involved in the development of the intervention however, as the host institution of the researcher (at the time), their name was mentioned on the website. In addition (and to the researcher's knowledge), the host institution did not hold affiliations with prestigious hospitals or universities.

5) The interventions for each group with sufficient details to allow replication, including how and when they were actually administered

5-i) Mention names, credential, affiliations of the developers, sponsors, and owners

Mention names, credential, affiliations of the developers, sponsors, and owners [6] (if authors/evaluators are owners or developer of the software, this needs to be declared in a "Conflict of interest" section or mentioned elsewhere in the manuscript).

	1	2	3	4	5	
subitem not at all important	○	○	○	●	○	essential

Your response is too large. Try shortening some answers.

Does your paper address subitem 5-i?

Copy and paste relevant sections from the manuscript (include quotes in quotation marks "like this" to indicate direct quotes from your manuscript), or elaborate on this item by providing additional information not in the ms, or briefly explain why the item is not applicable/relevant for your study

Yes - authors declare their ownership/evaluative role over the website in the "Conflicts of Interest" section of this paper: "Authors had an active role in the design, development and evaluation of the SleepWise intervention."

5-ii) Describe the history/development process

Describe the history/development process of the application and previous formative evaluations (e.g., focus groups, usability testing), as these will have an impact on adoption/use rates and help with interpreting results.

	1	2	3	4	5	
subitem not at all important	○	◉	○	○	○	essential

Clear selection

Your response is too large. Try shortening some answers.

Does your paper address subitem 5-ii?

Copy and paste relevant sections from the manuscript (include quotes in quotation marks "like this" to indicate direct quotes from your manuscript), or elaborate on this item by providing additional information not in the ms, or briefly explain why the item is not applicable/relevant for your study

This item is not wholly relevant/applicable to this paper as this paper reports on the feasibility testing of the intervention; the development process of the intervention is reported elsewhere however, a brief description of the intervention is provided in the paper:

"In brief, SleepWise consists of four weekly online (web-based) sessions, completed over a 5-week period. Each session is tunnelled (ie., participants work through a set number of pages), whilst additional ‘click-through’ options provide further information. Session 1 provides information about the importance of sleep for young people, and how eating patterns impact sleep. Session 2 is about physical activity and sleep quality, and sessions 3 and 4 are about the relationship between sleep and everyday habits and environments among young people (e.g., screen use, sleep environment etc).

Participants in this study were encouraged to use SleepWise to set weekly goals and complete a daily sleep diary. At the start of each weekly session, participants reviewed their goals from the previous week and were given the option to set new goals or continue with the same goals. At the end of each weekly session, participants had the option to complete quizzes and play educational games (see Multimedia Appendices 1 and 2). SleepWise also provides participants with optional sleep aiding tools such as sleep routine charts, mindfulness recordings, and information to external resources."

Your response is too large. Try shortening some answers.

5-iii) Revisions and updating

Revisions and updating. Clearly mention the date and/or version number of the application/intervention (and comparator, if applicable) evaluated, or describe whether the intervention underwent major changes during the evaluation process, or whether the development and/or content was “frozen” during the trial. Describe dynamic components such as news feeds or changing content which may have an impact on the replicability of the intervention (for unexpected events see item 3b).

	1	2	3	4	5	
subitem not at all important	○	◉	○	○	○	essential

Clear selection

Your response is too large. Try shortening some answers.

Does your paper address subitem 5-iii?

Copy and paste relevant sections from the manuscript (include quotes in quotation marks "like this" to indicate direct quotes from your manuscript), or elaborate on this item by providing additional information not in the ms, or briefly explain why the item is not applicable/relevant for your study

This item is not wholly relevant/applicable to this paper as this paper reports on the feasibility testing of the intervention; the development process of the intervention is reported elsewhere. The intervention did not undergo major changes during the trials in this paper. A brief description of the intervention is provided in the paper:

"In brief, SleepWise consists of four weekly online (web-based) sessions, completed over a 4-week period. Each session is tunnelled (ie., participants work through a set number of pages), whilst additional ‘click-through’ options provide further information. Session 1 provides information about the importance of sleep for young people, and how eating patterns impact sleep. Session 2 is about physical activity and sleep quality, and sessions 3 and 4 are about the relationship between sleep and everyday habits and environments among young people (e.g., screen use, sleep environment etc).

Participants in this study were encouraged to use SleepWise to set weekly goals and complete a daily sleep diary. At the start of each weekly session, participants reviewed their goals from the previous week and were given the option to set new goals or continue with the same goals. At the end of each weekly session, participants had the option to complete quizzes and play educational games (see Multimedia Appendices 1 and 2). SleepWise also provides participants with optional sleep aiding tools such as sleep routine charts, mindfulness recordings, and information to external resources."

Your response is too large. Try shortening some answers.

5-iv) Quality assurance methods

Provide information on quality assurance methods to ensure accuracy and quality of information provided [1], if applicable.

	1	2	3	4	5	
subitem not at all important	○	○	○	◉	○	essential

Clear selection

Your response is too large. Try shortening some answers.

Does your paper address subitem 5-iv?

Copy and paste relevant sections from the manuscript (include quotes in quotation marks "like this" to indicate direct quotes from your manuscript), or elaborate on this item by providing additional information not in the ms, or briefly explain why the item is not applicable/relevant for your study

Please see the next page for answer.

Your response is too large. Try shortening some answers.

Yes - the methods section details validated tools to collect participant data on sleep quality and acceptability of the intervention as well as user engagement as part of a process evaluation:

"The Pittsburgh Sleep Quality Index Short Form (PSQI-SF)

The PSQI-SF32 was used to measure sleep quality among participants. The PSQI-SF is a well-validated self-rated questionnaire that has been used widely in the adolescent population, to assess subjective sleep quality and disturbances, and the impact of poor sleep on functioning33–35. The PSQI-SF contains 13 questions and measures five dimensions: sleep latency, sleep duration, sleep efficiency, sleep disturbances, and daytime dysfunction. Items are rated from 0 (very good) to 3 (very bad)32. A total score greater than “4” is indicative of poor sleep quality32. Cronbach α for the five components of the PSQI-SF was .73 pre study and .52 at post study in trial 1, and .81 and .79 respectively in trial 2.

Acceptability E-Scale

Acceptability of the intervention was assessed using the 6-item Acceptability E-Scale36. This scale measures the extent to which users deemed the intervention to be acceptable. Items are rated from 1 (negative evaluation) to 5 (positive evaluation). Scores on each subscale were summed for a total acceptability score. Higher scores indicate a higher level of acceptance. This measure was slightly adapted (replacing “computer program” with “this website” and removing one item that was not relevant to this study (asked how helpful the program was for describing symptoms and quality of life). Cronbach α for the five items of this scale was .76 in Trial 1 and .66 in Trial 2

Specifically, to better understand participant engagement with the intervention, the quantitative component of the process evaluation evaluated participants’ engagement with each weekly session (time and pages viewed) and the number of goals and sleep logs completed across the intervention period, using SleepWise’s automatic data tracker."

Your response is too large. Try shortening some answers.

5-v) Ensure replicability by publishing the source code, and/or providing screenshots/screen-capture video, and/or providing flowcharts of the algorithms used

Ensure replicability by publishing the source code, and/or providing screenshots/screen-capture video, and/or providing flowcharts of the algorithms used. Replicability (i.e., other researchers should in principle be able to replicate the study) is a hallmark of scientific reporting.

	1	2	3	4	5	
subitem not at all important	○	◉	○	○	○	essential

Clear selection

Does your paper address subitem 5-v?

Copy and paste relevant sections from the manuscript (include quotes in quotation marks "like this" to indicate direct quotes from your manuscript), or elaborate on this item by providing additional information not in the ms, or briefly explain why the item is not applicable/relevant for your study

This item is not relevant/applicable to this paper as the study did not report on the development nor the replicability of the intervention and therefore this data was not published in this paper. The development process of the intervention is reported elsewhere outside of this paper.

Your response is too large. Try shortening some answers.

5-vi) Digital preservation

Digital preservation: Provide the URL of the application, but as the intervention is likely to change or disappear over the course of the years; also make sure the intervention is archived (Internet Archive, webcitation.org, and/or publishing the source code or screenshots/videos alongside the article). As pages behind login screens cannot be archived, consider creating demo pages which are accessible without login.

	1	2	3	4	5	
subitem not at all important	○	◉	○	○	○	essential

Clear selection

Does your paper address subitem 5-vi?

Copy and paste relevant sections from the manuscript (include quotes in quotation marks "like this" to indicate direct quotes from your manuscript), or elaborate on this item by providing additional information not in the ms, or briefly explain why the item is not applicable/relevant for your study

This item is not relevant/applicable to this paper as the study did not report on the development nor the replicability of the intervention and therefore this data is not included in this paper. The development process of the intervention is reported elsewhere outside of this paper.

Your response is too large. Try shortening some answers.

5-vii) Access

Access: Describe how participants accessed the application, in what setting/context, if they had to pay (or were paid) or not, whether they had to be a member of specific group. If known, describe how participants obtained “access to the platform and Internet” [1]. To ensure access for editors/reviewers/readers, consider to provide a “backdoor” login account or demo mode for reviewers/readers to explore the application (also important for archiving purposes, see vi).

	1	2	3	4	5	
subitem not at all important	○	○	◉	○	○	essential

Clear selection

Does your paper address subitem 5-vii? *

Copy and paste relevant sections from the manuscript (include quotes in quotation marks "like this" to indicate direct quotes from your manuscript), or elaborate on this item by providing additional information not in the ms, or briefly explain why the item is not applicable/relevant for your study

This item is not wholly relevant/applicable to this paper as instructions to join/access the intervention were explicitly outlined in an email to the intervention participants that contained a link to accessing the intervention and therefore this is not detailed in the paper. Participants had to option to get in touch with the researcher if they experienced problems accessing the intervention. A brief description of this method of access to the intervention is included in the methods section of this paper: "Participants in the intervention group were emailed weekly links to access the (free of charge) intervention and with instructions, were asked to work through it for 4 to 5 weeks.".

5-viii) Mode of delivery, features/functionalities/components of the intervention and comparator, and the theoretical framework

Describe mode of delivery, features/functionalities/components of the intervention and comparator, and the theoretical framework [6] used to design them (instructional strategy [1], behaviour change techniques, persuasive features, etc., see e.g., [7, 8] for terminology). This includes an in-depth description of the content (including where it is coming from and who developed it) [1]," whether [and how] it is tailored to individual circumstances and allows users to track their progress and receive feedback" [6]. This also includes a description of communication delivery channels and – if computer-mediated communication is a component – whether communication was synchronous or asynchronous [6]. It also includes information on presentation strategies [1], including page design principles, average amount of text on pages, presence of hyperlinks to other resources, etc. [1].

	1	2	3	4	5	
subitem not at all important	(●)	( )	( )	( )	( )	essential

Clear selection

Your response is too large. Try shortening some answers.

Does your paper address subitem 5-viii? *

Copy and paste relevant sections from the manuscript (include quotes in quotation marks "like this" to indicate direct quotes from your manuscript), or elaborate on this item by providing additional information not in the ms, or briefly explain why the item is not applicable/relevant for your study

This item is not relevant/applicable to this paper as the study did not report on the development and therefore the specific features/functionalities/components of the intervention or the theoretical framework. This information is therefore not included in this paper. The paper does however report on the comparator: "Those allocated to the control group continued to receive standard education but were given access to the intervention at the end of the study if they wished...Participants in the intervention group were emailed weekly links to access the (free of charge) intervention and with instructions, were asked to work through it for 4 to 5 weeks."

5-ix) Describe use parameters

Describe use parameters (e.g., intended “doses” and optimal timing for use). Clarify what instructions or recommendations were given to the user, e.g., regarding timing, frequency, heaviness of use, if any, or was the intervention used ad libitum.

	1	2	3	4	5	
subitem not at all important	○	○	◉	○	○	essential

Clear selection

Your response is too large. Try shortening some answers.

Does your paper address subitem 5-ix?

Copy and paste relevant sections from the manuscript (include quotes in quotation marks "like this" to indicate direct quotes from your manuscript), or elaborate on this item by providing additional information not in the ms, or briefly explain why the item is not applicable/relevant for your study

Yes. Detailed instructions were included in the email sent to participants in the intervention group: "Participants in the intervention group were emailed weekly links to access the (free of charge) intervention and with instructions, were asked to work through it for 4 to 5 weeks. Intervention participants were also sent weekly email and text reminders, reminding them about their set goals and logging their sleep patterns."

5-x) Clarify the level of human involvement

Clarify the level of human involvement (care providers or health professionals, also technical assistance) in the e-intervention or as co-intervention (detail number and expertise of professionals involved, if any, as well as “type of assistance offered, the timing and frequency of the support, how it is initiated, and the medium by which the assistance is delivered”. It may be necessary to distinguish between the level of human involvement required for the trial, and the level of human involvement required for a routine application outside of a RCT setting (discuss under item 21 – generalizability).

	1	2	3	4	5	
subitem not at all important	○	◉	○	○	○	essential

Clear selection

Your response is too large. Try shortening some answers.

Does your paper address subitem 5-x?

Copy and paste relevant sections from the manuscript (include quotes in quotation marks "like this" to indicate direct quotes from your manuscript), or elaborate on this item by providing additional information not in the ms, or briefly explain why the item is not applicable/relevant for your study

This item is not wholly relevant/applicable to this paper as there was no human involvement in the e-intervention or included as an co-intervention. However, participants were sent reminders to complete self-monitoring tasks (such as keeping a sleep diary) and had the option to opt out of reminders if they wished: "Participants in the intervention group were emailed weekly links to access the (free of charge) intervention and with instructions, were asked to work through it for 4 to 5 weeks. Intervention participants were also sent weekly email and text reminders, reminding them about their set goals and logging their sleep patterns. Participants had the option to opt out of these reminders if they wished."

5-xi) Report any prompts/reminders used

Report any prompts/reminders used: Clarify if there were prompts (letters, emails, phone calls, SMS) to use the application, what triggered them, frequency etc. It may be necessary to distinguish between the level of prompts/reminders required for the trial, and the level of prompts/reminders for a routine application outside of a RCT setting (discuss under item 21 – generalizability).

	1	2	3	4	5	
subitem not at all important	○	○	○	○	◉	essential

Your response is too large. Try shortening some answers.

Does your paper address subitem 5-xi? *

Copy and paste relevant sections from the manuscript (include quotes in quotation marks "like this" to indicate direct quotes from your manuscript), or elaborate on this item by providing additional information not in the ms, or briefly explain why the item is not applicable/relevant for your study

Yes - "Participants in the intervention group were emailed weekly links to access the (free of charge) intervention and with instructions, were asked to work through it for 4 to 5 weeks. Intervention participants were also sent weekly email and text reminders, reminding them about their set goals and logging their sleep patterns. Participants had the option to opt out of these reminders if they wished."

5-xii) Describe any co-interventions (incl. training/support)

Describe any co-interventions (incl. training/support): Clearly state any interventions that are provided in addition to the targeted eHealth intervention, as ehealth intervention may not be designed as stand-alone intervention. This includes training sessions and support [1]. It may be necessary to distinguish between the level of training required for the trial, and the level of training for a routine application outside of a RCT setting (discuss under item 21 – generalizability.

	1	2	3	4	5	
subitem not at all important	◉	○	○	○	○	essential

Clear selection

Your response is too large. Try shortening some answers.

Does your paper address subitem 5-xii? *

Copy and paste relevant sections from the manuscript (include quotes in quotation marks "like this" to indicate direct quotes from your manuscript), or elaborate on this item by providing additional information not in the ms, or briefly explain why the item is not applicable/relevant for your study

This item is not relevant/applicable to this paper as the intervention was standalone i.e, the were no co-interventions. Technical support (e.g., resending link/s) was offered to participants by the researcher if they experienced problems accessing the intervention; however, this was only technical support and not a co-intervention or an addition to the intervention. This was deatiled in the email intevrention participants received in the intervention group: "Participants in the intervention group were emailed weekly links to access the (free of charge) intervention and with instructions, were asked to work through it for 4 to 5 weeks".

6a) Completely defined pre-specified primary and secondary outcome measures, including how and when they were assessed

Your response is too large. Try shortening some answers.

Does your paper address CONSORT subitem 6a? *

Copy and paste relevant sections from the manuscript (include quotes in quotation marks "like this" to indicate direct quotes from your manuscript), or elaborate on this item by providing additional information not in the ms, or briefly explain why the item is not applicable/relevant for your study

Yes - "...participants completed a baseline measure questionnaire about their sleep quality via Qualtrics (an online data administration software). Participants in both groups were asked to complete this measure at baseline and at 5 weeks post study... Intervention participants were asked additional questions about the intervention’s acceptability at the post study 5-week stage."

6a-i) Online questionnaires: describe if they were validated for online use and apply CHERRIES items to describe how the questionnaires were designed/deployed

If outcomes were obtained through online questionnaires, describe if they were validated for online use and apply CHERRIES items to describe how the questionnaires were designed/deployed [9].

	1	2	3	4	5	
subitem not at all important	○	○	◉	○	○	essential

Clear selection

Your response is too large. Try shortening some answers.

Does your paper address subitem 6a-i?

Copy and paste relevant sections from manuscript text

Yes - "The Pittsburgh Sleep Quality Index Short Form (PSQI-SF)
The PSQI-SF32 was used to measure sleep quality among participants. The PSQI-SF is a well-validated self-rated questionnaire that has been used widely in the adolescent population, to assess subjective sleep quality and disturbances, and the impact of poor sleep on functioning33–35. The PSQI-SF contains 13 questions and measures five dimensions: sleep latency, sleep duration, sleep efficiency, sleep disturbances, and daytime dysfunction. Items are rated from 0 (very good) to 3 (very bad)32. A total score greater than “4” is indicative of poor sleep quality32. Cronbach α for the five components of the PSQI-SF was .73 pre study and .52 at post study in trial 1, and .81 and .79 respectively in trial 2.

Acceptability E-Scale
Acceptability of the intervention was assessed using the 6-item Acceptability E-Scale36. This scale measures the extent to which users deemed the intervention to be acceptable. Items are rated from 1 (negative evaluation) to 5 (positive evaluation). Scores on each subscale were summed for a total acceptability score. Higher scores indicate a higher level of acceptance. This measure was slightly adapted (replacing “computer program” with “this website” and removing one item that was not relevant to this study (asked how helpful the program was for describing symptoms and quality of life). Cronbach α for the five items of this scale was .76 in Trial 1 and .66 in Trial 2."

Your response is too large. Try shortening some answers.

6a-ii) Describe whether and how “use” (including intensity of use/dosage) was defined/measured/monitored

Describe whether and how “use” (including intensity of use/dosage) was defined/measured/monitored (logins, logfile analysis, etc.). Use/adoption metrics are important process outcomes that should be reported in any ehealth trial.

	1	2	3	4	5	
subitem not at all important	○	○	○	◉	○	essential

Clear selection

Does your paper address subitem 6a-ii?

Copy and paste relevant sections from manuscript text

Yes - "To better understand participant engagement with the intervention, the quantitative component of the process evaluation evaluated participants’ engagement with each weekly session (time and pages viewed) and the number of goals and sleep logs completed across the intervention period, using SleepWise’s automatic data tracker...Data about the date, time, frequency of participants’ weekly goals, sleep logs, and pages visited was automatically tracked by the SleepWise website and used as a measure of participant engagement with the intervention."

Your response is too large. Try shortening some answers.

6a-iii) Describe whether, how, and when qualitative feedback from participants was obtained

Describe whether, how, and when qualitative feedback from participants was obtained (e.g., through emails, feedback forms, interviews, focus groups).

	1	2	3	4	5	
subitem not at all important	○	○	○	○	◉	essential

Clear selection

Does your paper address subitem 6a-iii?

Copy and paste relevant sections from manuscript text

Yes- "The qualitative component of the process evaluation explored participants' experiences with SleepWise, via qualitative follow-up interviews. Participants from both trials across both the intervention and control groups were contacted between 5 to 8 weeks post study via phone-call interviews...Interviews were audio recorded and lasted for approximately 20 to 30 minutes."

6b) Any changes to trial outcomes after the trial commenced, with reasons

Your response is too large. Try shortening some answers.

Does your paper address CONSORT subitem 6b? *

Copy and paste relevant sections from the manuscript (include quotes in quotation marks "like this" to indicate direct quotes from your manuscript), or elaborate on this item by providing additional information not in the ms, or briefly explain why the item is not applicable/relevant for your study

This item is not relevant or applicable to this paper as no changes to trial outcomes were made after the trial commenced.

7a) How sample size was determined

NPT: When applicable, details of whether and how the clustering by care provides or centers was addressed

7a-i) Describe whether and how expected attrition was taken into account when calculating the sample size

Describe whether and how expected attrition was taken into account when calculating the sample size.

	1	2	3	4	5	
subitem not at all important	○	○	○	○	●	essential

Clear selection

Your response is too large. Try shortening some answers.

Does your paper address subitem 7a-i?

Copy and paste relevant sections from manuscript title (include quotes in quotation marks "like this" to indicate direct quotes from your manuscript), or elaborate on this item by providing additional information not in the ms, or briefly explain why the item is not applicable/relevant for your study

Yes - "A prior power analysis suggested a participant sample of 100 was required based on the assumption that it allowed for a 40 to 50% dropout or loss to follow-up, which is common in internet-based research. This resulted in an overall sample of at least 50 participant per trial (25 to intervention and 25 in control)."

7b) When applicable, explanation of any interim analyses and stopping guidelines

Does your paper address CONSORT subitem 7b? *

Copy and paste relevant sections from the manuscript (include quotes in quotation marks "like this" to indicate direct quotes from your manuscript), or elaborate on this item by providing additional information not in the ms, or briefly explain why the item is not applicable/relevant for your study

This item is not relevant to this paper as no interim analyses or stopping guidelines were included in the study.

Your response is too large. Try shortening some answers.

Does your paper address CONSORT subitem 8a? *

Copy and paste relevant sections from the manuscript (include quotes in quotation marks "like this" to indicate direct quotes from your manuscript), or elaborate on this item by providing additional information not in the ms, or briefly explain why the item is not applicable/relevant for your study

Yes - "Participants were randomly allocated to the intervention or control group using an online randomization generator (Randomisation.com)."

8b) Type of randomisation; details of any restriction (such as blocking and block size)

Does your paper address CONSORT subitem 8b? *

Copy and paste relevant sections from the manuscript (include quotes in quotation marks "like this" to indicate direct quotes from your manuscript), or elaborate on this item by providing additional information not in the ms, or briefly explain why the item is not applicable/relevant for your study

Yes - "Participants were randomly allocated to the intervention or control group using an online randomization generator (Randomisation.com). "

Your response is too large. Try shortening some answers.

9) Mechanism used to implement the random allocation sequence (such as sequentially numbered containers), describing any steps taken to conceal the sequence until interventions were assigned

Does your paper address CONSORT subitem 9? *

Copy and paste relevant sections from the manuscript (include quotes in quotation marks "like this" to indicate direct quotes from your manuscript), or elaborate on this item by providing additional information not in the ms, or briefly explain why the item is not applicable/relevant for your study

Yes - "Participants were randomly allocated to the intervention or control group using an online randomization generator (Randomisation.com). "

10) Who generated the random allocation sequence, who enrolled participants, and who assigned participants to interventions

Your response is too large. Try shortening some answers.

Does your paper address CONSORT subitem 10? *

Copy and paste relevant sections from the manuscript (include quotes in quotation marks "like this" to indicate direct quotes from your manuscript), or elaborate on this item by providing additional information not in the ms, or briefly explain why the item is not applicable/relevant for your study

Yes - "Participants were randomly allocated to the intervention or control group using an online randomization generator (Randomisation.com). "

11a) If done, who was blinded after assignment to interventions (for example, participants, care providers, those assessing outcomes) and how

NPT: Whether or not administering co-interventions were blinded to group assignment

11a-i) Specify who was blinded, and who wasn't

Specify who was blinded, and who wasn't. Usually, in web-based trials it is not possible to blind the participants [1, 3] (this should be clearly acknowledged), but it may be possible to blind outcome assessors, those doing data analysis or those administering co-interventions (if any).

	1	2	3	4	5	
subitem not at all important	○	○	◉	○	○	essential

Clear selection

Your response is too large. Try shortening some answers.

Does your paper address subitem 11a-i? *

Copy and paste relevant sections from the manuscript (include quotes in quotation marks "like this" to indicate direct quotes from your manuscript), or elaborate on this item by providing additional information not in the ms, or briefly explain why the item is not applicable/relevant for your study

Yes - "Neither participants nor the researcher were blinded to allocation as it is often not possible to blind participants in web-based trials, and the researcher was the core member undertaking this research and it was not possible for them to be blinded."

11a-ii) Discuss e.g., whether participants knew which intervention was the “intervention of interest” and which one was the “comparator”

Informed consent procedures (4a-ii) can create biases and certain expectations - discuss e.g., whether participants knew which intervention was the “intervention of interest” and which one was the “comparator”.

	1	2	3	4	5	
subitem not at all important	○	◉	○	○	○	essential

Clear selection

Your response is too large. Try shortening some answers.

Does your paper address subitem 11a-ii?

Copy and paste relevant sections from the manuscript (include quotes in quotation marks "like this" to indicate direct quotes from your manuscript), or elaborate on this item by providing additional information not in the ms, or briefly explain why the item is not applicable/relevant for your study

Yes, this was made explicit to participants in the study's information sheet, consent form and emails revealing allocation: "Allocation was revealed to participants in an email containing information to access the intervention (intervention group) or to continue with normal education (control group). Control participants continued to receive standard education but were informed about the importance of their role in the study, and given access to the intervention at the end of the study if they wished."

11b) If relevant, description of the similarity of interventions

(this item is usually not relevant for ehealth trials as it refers to similarity of a placebo or sham intervention to a active medication/intervention)

Does your paper address CONSORT subitem 11b? *

Copy and paste relevant sections from the manuscript (include quotes in quotation marks "like this" to indicate direct quotes from your manuscript), or elaborate on this item by providing additional information not in the ms, or briefly explain why the item is not applicable/relevant for your study

This item is not relevant or applicable to this paper as there was only one standalone intervention (being compared to usual education) and no placebos or sham intervention was included in this study.

12a) Statistical methods used to compare groups for primary and secondary outcomes

NPT: When applicable, details of whether and how the clustering by care providers or centers was addressed

Does your paper address CONSORT subitem 12a? *

Copy and paste relevant sections from the manuscript (include quotes in quotation marks "like this" to indicate direct quotes from your manuscript), or elaborate on this item by providing additional information not in the ms, or briefly explain why the item is not applicable/relevant for your study

Yes - "Descriptive statistics were used to illustrate participant characteristics. An exploratory effect size analysis was undertaken to explore initial indicators of SleepWise on the primary outcome of sleep quality. An exploratory effect size, using Cohen d, was calculated for the mean changes in sleep quality (PSQI-SF) for intervention completers by subtracting participants' follow-up sleep quality mean from their baseline sleep quality mean (mean difference) in the two study groups by using the SPSS software (Version 26). Cohen d is deemed more suitable for determining the effect of an intervention in contrast to r, which is more appropriate for evaluating correlations between variables,"

Your response is too large. Try shortening some answers.

12a-i) Imputation techniques to deal with attrition / missing values

Imputation techniques to deal with attrition / missing values: Not all participants will use the intervention/comparator as intended and attrition is typically high in ehealth trials. Specify how participants who did not use the application or dropped out from the trial were treated in the statistical analysis (a complete case analysis is strongly discouraged, and simple imputation techniques such as LOCF may also be problematic [4]).

	1	2	3	4	5	
subitem not at all important	◉	○	○	○	○	essential

Clear selection

Does your paper address subitem 12a-i? *

Copy and paste relevant sections from the manuscript (include quotes in quotation marks "like this" to indicate direct quotes from your manuscript), or elaborate on this item by providing additional information not in the ms, or briefly explain why the item is not applicable/relevant for your study

This item is not relevant or applicable to this paper as all study questionnaires were compulsory and therefore if participants did not complete questionnaires their data could not be included/analysed.

12b) Methods for additional analyses, such as subgroup analyses and adjusted

Your response is too large. Try shortening some answers.

Does your paper address CONSORT subitem 12b? *

Copy and paste relevant sections from the manuscript (include quotes in quotation marks "like this" to indicate direct quotes from your manuscript), or elaborate on this item by providing additional information not in the ms, or briefly explain why the item is not applicable/relevant for your study

This item is not relevant or applicable to this paper as no additional analyses were undertaken in the study.

X26) REB/IRB Approval and Ethical Considerations [recommended as subheading under "Methods"] (not a CONSORT item)

X26-i) Comment on ethics committee approval

	1	2	3	4	5	
subitem not at all important	○	○	○	○	◉	essential

Clear selection

Your response is too large. Try shortening some answers.

Does your paper address subitem X26-i?

Copy and paste relevant sections from the manuscript (include quotes in quotation marks "like this" to indicate direct quotes from your manuscript), or elaborate on this item by providing additional information not in the ms, or briefly explain why the item is not applicable/relevant for your study

Yes - "Ethical approval for the study was received from the University's ethics committee (XXX), and the study was undertaken in accordance with the Helsinki Declaration"

x26-ii) Outline informed consent procedures

Outline informed consent procedures e.g., if consent was obtained offline or online (how? Checkbox, etc.?), and what information was provided (see 4a-ii). See [6] for some items to be included in informed consent documents.

	1	2	3	4	5	
subitem not at all important	○	○	○	○	●	essential

Clear selection

Your response is too large. Try shortening some answers.

Does your paper address subitem X26-ii?

Copy and paste relevant sections from the manuscript (include quotes in quotation marks "like this" to indicate direct quotes from your manuscript), or elaborate on this item by providing additional information not in the ms, or briefly explain why the item is not applicable/relevant for your study

Yes - "Adolescents who were interested in partaking in the study collected consent and information sheets from the study facilitator (ie., site staff). Signed consent forms were then collected by the researcher from each site. Parental consent was not required for participants aged 16 years and over, as per the British Psychological Society's (BPS) ethical guidelines."

X26-iii) Safety and security procedures

Safety and security procedures, incl. privacy considerations, and any steps taken to reduce the likelihood or detection of harm (e.g., education and training, availability of a hotline)

	1	2	3	4	5	
subitem not at all important	○	○	○	○	●	essential

Clear selection

Your response is too large. Try shortening some answers.

Does your paper address subitem X26-iii?

Copy and paste relevant sections from the manuscript (include quotes in quotation marks "like this" to indicate direct quotes from your manuscript), or elaborate on this item by providing additional information not in the ms, or briefly explain why the item is not applicable/relevant for your study

Yes, - this is detailed in the study's participant information sheet and therefore not detailed in the paper however it is mentioned in the paper that this study followed ethical guidelines set out by the British Psychological Society and received ethical approval: "Ethical approval for the study was received from the University's ethics committee (XXX), and the study was undertaken in accordance with the Helsinki Declaration... Parental consent was not required for participants aged 16 years and over, as per the British Psychological Society's (BPS) ethical guidelines."

RESULTS

13a) For each group, the numbers of participants who were randomly assigned, received intended treatment, and were analysed for the primary outcome

NPT: The number of care providers or centers performing the intervention in each group and the number of patients treated by each care provider in each center

Your response is too large. Try shortening some answers.

Does your paper address CONSORT subitem 13a? *

Copy and paste relevant sections from the manuscript (include quotes in quotation marks "like this" to indicate direct quotes from your manuscript), or elaborate on this item by providing additional information not in the ms, or briefly explain why the item is not applicable/relevant for your study

Yes - this item is reflected in a PRISMA flow diagram: "Figures 1 and 2 show the recruitment, attrition, and completion process in Trial 1 and Trial 2".

13b) For each group, losses and exclusions after randomisation, together with reasons

Does your paper address CONSORT subitem 13b? (NOTE: Preferably, this is shown in a CONSORT flow diagram) *

Copy and paste relevant sections from the manuscript (include quotes in quotation marks "like this" to indicate direct quotes from your manuscript), or elaborate on this item by providing additional information not in the ms, or briefly explain why the item is not applicable/relevant for your study

Yes - losses and exclusions after randomisation are shown in Figures 1 and 2 (PRISMA flow diagrams) under the Results section.

Your response is too large. Try shortening some answers.

13b-i) Attrition diagram

Strongly recommended: An attrition diagram (e.g., proportion of participants still logging in or using the intervention/comparator in each group plotted over time, similar to a survival curve) or other figures or tables demonstrating usage/dose/engagement.

	1	2	3	4	5	
subitem not at all important	○	○	○	◉	○	essential

Clear selection

Does your paper address subitem 13b-i?

Copy and paste relevant sections from the manuscript or cite the figure number if applicable (include quotes in quotation marks "like this" to indicate direct quotes from your manuscript), or elaborate on this item by providing additional information not in the ms, or briefly explain why the item is not applicable/relevant for your study

Yes - participant engagement with the intervention is demonstrated in Figures 3 and 4.

14a) Dates defining the periods of recruitment and follow-up

Your response is too large. Try shortening some answers.

Does your paper address CONSORT subitem 14a? *

Copy and paste relevant sections from the manuscript (include quotes in quotation marks "like this" to indicate direct quotes from your manuscript), or elaborate on this item by providing additional information not in the ms, or briefly explain why the item is not applicable/relevant for your study

Yes - "Trial 1 recruitment took place from November 2018 to May 2019...Trial 2 recruitment took place from May 2019 to December 2019...".

14a-i) Indicate if critical “secular events” fell into the study period

Indicate if critical “secular events” fell into the study period, e.g., significant changes in Internet resources available or “changes in computer hardware or Internet delivery resources”

	1	2	3	4	5	
subitem not at all important	(●)	( )	( )	( )	( )	essential

Clear selection

Your response is too large. Try shortening some answers.

Does your paper address subitem 14a-i?

Copy and paste relevant sections from the manuscript (include quotes in quotation marks "like this" to indicate direct quotes from your manuscript), or elaborate on this item by providing additional information not in the ms, or briefly explain why the item is not applicable/relevant for your study

This item is not relevant to this paper as no critical secular events fell into the study period.

14b) Why the trial ended or was stopped (early)

Does your paper address CONSORT subitem 14b? *

Copy and paste relevant sections from the manuscript (include quotes in quotation marks "like this" to indicate direct quotes from your manuscript), or elaborate on this item by providing additional information not in the ms, or briefly explain why the item is not applicable/relevant for your study

This item is not relevant to this paper as the trial did not end or stop early.

15) A table showing baseline demographic and clinical characteristics for each group

NPT: When applicable, a description of care providers (case volume, qualification,

Your response is too large. Try shortening some answers.

Does your paper address CONSORT subitem 15? *

Copy and paste relevant sections from the manuscript (include quotes in quotation marks "like this" to indicate direct quotes from your manuscript), or elaborate on this item by providing additional information not in the ms, or briefly explain why the item is not applicable/relevant for your study

Yes - this is demonstrated in Table 2 named "Participant Demographics in Each Trial".

15-i) Report demographics associated with digital divide issues

In ehealth trials it is particularly important to report demographics associated with digital divide issues, such as age, education, gender, social-economic status, computer/Internet/ehealth literacy of the participants, if known.

	1	2	3	4	5	
subitem not at all important	○	○	◉	○	○	essential

Clear selection

Your response is too large. Try shortening some answers.

Does your paper address subitem 15-i? *

Copy and paste relevant sections from the manuscript (include quotes in quotation marks "like this" to indicate direct quotes from your manuscript), or elaborate on this item by providing additional information not in the ms, or briefly explain why the item is not applicable/relevant for your study

Yes - this is demonstrated in Table 2.

16) For each group, number of participants (denominator) included in each analysis and whether the analysis was by original assigned groups

16-i) Report multiple “denominators” and provide definitions

Report multiple “denominators” and provide definitions: Report N’s (and effect sizes) “across a range of study participation [and use] thresholds” [1], e.g., N exposed, N consented, N used more than x times, N used more than y weeks, N participants “used” the intervention/comparator at specific pre-defined time points of interest (in absolute and relative numbers per group). Always clearly define “use” of the intervention.

	1	2	3	4	5	
subitem not at all important	(•)	○	○	○	○	essential

Clear selection

Your response is too large. Try shortening some answers.

Does your paper address subitem 16-i? *

Copy and paste relevant sections from the manuscript (include quotes in quotation marks "like this" to indicate direct quotes from your manuscript), or elaborate on this item by providing additional information not in the ms, or briefly explain why the item is not applicable/relevant for your study

This item is partly met in this paper as participant engagement with the intervention across the four week period was tracked and presented in the paper in Figures 3 and 4 under section "Participant engagement with SleepWise" .

16-ii) Primary analysis should be intent-to-treat

Primary analysis should be intent-to-treat, secondary analyses could include comparing only “users”, with the appropriate caveats that this is no longer a randomized sample (see 18-i).

	1	2	3	4	5	
subitem not at all important	(●)	( )	( )	( )	( )	essential

Clear selection

Your response is too large. Try shortening some answers.

Does your paper address subitem 16-ii?

Copy and paste relevant sections from the manuscript (include quotes in quotation marks "like this" to indicate direct quotes from your manuscript), or elaborate on this item by providing additional information not in the ms, or briefly explain why the item is not applicable/relevant for your study

No - this analysis was not carried out as it was not in line with the aims of the paper (the study was a feasibility study i.e., only interested in preliminary effect sizes and therefore not definitive/effectiveness testing of the intervention).

17a) For each primary and secondary outcome, results for each group, and the estimated effect size and its precision (such as 95% confidence interval)

Does your paper address CONSORT subitem 17a? *

Copy and paste relevant sections from the manuscript (include quotes in quotation marks "like this" to indicate direct quotes from your manuscript), or elaborate on this item by providing additional information not in the ms, or briefly explain why the item is not applicable/relevant for your study

Yes - this is reported for each trial under section titled "Exploratory Effect Sizes and Acceptability".

Your response is too large. Try shortening some answers.

17a-i) Presentation of process outcomes such as metrics of use and intensity of use

In addition to primary/secondary (clinical) outcomes, the presentation of process outcomes such as metrics of use and intensity of use (dose, exposure) and their operational definitions is critical. This does not only refer to metrics of attrition (13-b) (often a binary variable), but also to more continuous exposure metrics such as “average session length”. These must be accompanied by a technical description how a metric like a “session” is defined (e.g., timeout after idle time) [1] (report under item 6a).

	1	2	3	4	5	
subitem not at all important	○	○	◉	○	○	essential

Clear selection

Does your paper address subitem 17a-i?

Copy and paste relevant sections from the manuscript (include quotes in quotation marks "like this" to indicate direct quotes from your manuscript), or elaborate on this item by providing additional information not in the ms, or briefly explain why the item is not applicable/relevant for your study

Yes - this is desmonstaed under section titled "Participant Engagement with SleepWise" and visually illustrated in Figures 3 and 4.

Your response is too large. Try shortening some answers.

17b) For binary outcomes, presentation of both absolute and relative effect sizes is recommended

Does your paper address CONSORT subitem 17b? *

Copy and paste relevant sections from the manuscript (include quotes in quotation marks "like this" to indicate direct quotes from your manuscript), or elaborate on this item by providing additional information not in the ms, or briefly explain why the item is not applicable/relevant for your study

No - this analysis was not carried out as it was not in line with the aims of the study. The study was a feasibility study and only interested in preliminary effect sizes on sleep quality and acceptability of the intervention. This is reported for each trial under section titled "Exploratory Effect Sizes and Acceptability".

18) Results of any other analyses performed, including subgroup analyses and adjusted analyses, distinguishing pre-specified from exploratory

Your response is too large. Try shortening some answers.

Does your paper address CONSORT subitem 18? *

Copy and paste relevant sections from the manuscript (include quotes in quotation marks "like this" to indicate direct quotes from your manuscript), or elaborate on this item by providing additional information not in the ms, or briefly explain why the item is not applicable/relevant for your study

No - this analysis was not carried out as it was not in line with the aims of the study. The study was a feasibility study and only interested in preliminary effect sizes on sleep quality and acceptability of the intervention. This is reported for each trial under section titled "Exploratory Effect Sizes and Acceptability".

18-i) Subgroup analysis of comparing only users

A subgroup analysis of comparing only users is not uncommon in ehealth trials, but if done, it must be stressed that this is a self-selected sample and no longer an unbiased sample from a randomized trial (see 16-iii).

	1	2	3	4	5	
subitem not at all important	◉	○	○	○	○	essential

Clear selection

Your response is too large. Try shortening some answers.

Does your paper address subitem 18-i?

Copy and paste relevant sections from the manuscript (include quotes in quotation marks "like this" to indicate direct quotes from your manuscript), or elaborate on this item by providing additional information not in the ms, or briefly explain why the item is not applicable/relevant for your study

No - this analysis was not carried out as it was not in line with the aims of the study. The study was a feasibility study with a with a small sample size, interested only in preliminary effect sizes on sleep quality and acceptability of the intervention, among participants who continued to use the intervention across the designated period.

19) All important harms or unintended effects in each group

(for specific guidance see CONSORT for harms)

Does your paper address CONSORT subitem 19? *

Copy and paste relevant sections from the manuscript (include quotes in quotation marks "like this" to indicate direct quotes from your manuscript), or elaborate on this item by providing additional information not in the ms, or briefly explain why the item is not applicable/relevant for your study

No - no harm or unintended effects were found for any groups in this study and therefore not reported.

Your response is too large. Try shortening some answers.

19-i) Include privacy breaches, technical problems

Include privacy breaches, technical problems. This does not only include physical “harm” to participants, but also incidents such as perceived or real privacy breaches [1], technical problems, and other unexpected/unintended incidents. “Unintended effects” also includes unintended positive effects [2].

	1	2	3	4	5	
subitem not at all important	○	○	○	◉	○	essential

Clear selection

Does your paper address subitem 19-i?

Copy and paste relevant sections from the manuscript (include quotes in quotation marks "like this" to indicate direct quotes from your manuscript), or elaborate on this item by providing additional information not in the ms, or briefly explain why the item is not applicable/relevant for your study

No - no privacy breacher or technical problem were reported.

Your response is too large. Try shortening some answers.

19-ii) Include qualitative feedback from participants or observations from staff/researchers

Include qualitative feedback from participants or observations from staff/researchers, if available, on strengths and shortcomings of the application, especially if they point to unintended/unexpected effects or uses. This includes (if available) reasons for why people did or did not use the application as intended by the developers.

	1	2	3	4	5	
subitem not at all important	○	○	○	○	◉	essential

Clear selection

Does your paper address subitem 19-ii?

Copy and paste relevant sections from the manuscript (include quotes in quotation marks "like this" to indicate direct quotes from your manuscript), or elaborate on this item by providing additional information not in the ms, or briefly explain why the item is not applicable/relevant for your study

Yes - this is reported under section of the paper titled "Qualitative Findings".

DISCUSSION

Your response is too large. Try shortening some answers.

22) Interpretation consistent with results, balancing benefits and harms, and considering other relevant evidence

NPT: In addition, take into account the choice of the comparator, lack of or partial blinding, and unequal expertise of care providers or centers in each group

22-i) Restate study questions and summarize the answers suggested by the data, starting with primary outcomes and process outcomes (use)

Restate study questions and summarize the answers suggested by the data, starting with primary outcomes and process outcomes (use).

	1	2	3	4	5	
subitem not at all important	○	○	○	○	◉	essential

Clear selection

Does your paper address subitem 22-i? *

Copy and paste relevant sections from the manuscript (include quotes in quotation marks "like this" to indicate direct quotes from your manuscript), or elaborate on this item by providing additional information not in the ms, or briefly explain why the item is not applicable/relevant for your study

Yes - this is demonstrated in the first paragraph of the Discussion section.

Your response is too large. Try shortening some answers.

22-ii) Highlight unanswered new questions, suggest future research

Highlight unanswered new questions, suggest future research.

	1	2	3	4	5	
subitem not at all important	○	○	○	○	◉	essential

Clear selection

Your response is too large. Try shortening some answers.

Does your paper address subitem 22-ii?

Copy and paste relevant sections from the manuscript (include quotes in quotation marks "like this" to indicate direct quotes from your manuscript), or elaborate on this item by providing additional information not in the ms, or briefly explain why the item is not applicable/relevant for your study

Yes - this is highlighted in the Discussion section - for example "...personalized and flexible approaches to encouraging behavior change among adolescents may also have the added benefit of reflecting users' specific needs and allow adaptive and targeted communication. With adolescents strongly favouring more personalized and flexible approaches to intervention delivery, it is not surprising that innovative interventions - such as interactive digitalized sleep interventions - could replace one-off activity monitors and sleep logs, which may even decrease clinical time, and increase availability and accessibility to treatment options for adolescents with sleep-related problems. In addition to personalization and flexibility, sleep interventions should align with priorities and personal values of adolescents. For example, adolescent sleep can be impacted by increasing pressures to maintain social relationships (eg see Maume44) and homework demands (eg., see Norland et al.50), making it more difficult to manage health behaviours, including sleep. Given that adolescents favoured bite-sized education (eg., short weekly sessions), promoting short interactive digital communications via platforms such as SleepWise, could provide acceptable, time-efficient, and potentially efficacious solutions to encouraging and engaging adolescents with sleep behaviour change. Indeed, it is not surprising that online platforms are already being utilised to communicate health messages to adolescents (eg., see Yonker et al.51). To this end, it is clear that novel approaches to intervention development and delivery can help improve and maximise sleep health among adolescents, thus interventions like SleepWise warrant further exploration via a main trial of effectiveness."

Your response is too large. Try shortening some answers.

20) Trial limitations, addressing sources of potential bias, imprecision, and, if relevant, multiplicity of analyses

20-i) Typical limitations in ehealth trials

Typical limitations in ehealth trials: Participants in ehealth trials are rarely blinded. Ehealth trials often look at a multiplicity of outcomes, increasing risk for a Type I error. Discuss biases due to non-use of the intervention/usability issues, biases through informed consent procedures, unexpected events.

	1	2	3	4	5	
subitem not at all important	○	○	○	○	◉	essential

Clear selection

Your response is too large. Try shortening some answers.

Does your paper address subitem 20-i? *

Copy and paste relevant sections from the manuscript (include quotes in quotation marks "like this" to indicate direct quotes from your manuscript), or elaborate on this item by providing additional information not in the ms, or briefly explain why the item is not applicable/relevant for your study

While typical limitations in ehealth trials were not wholly covered in this paper, limitations of the current study were, as highlighted under the Discussion section of the paper - for example: "Limitations of this study include the lack of an active control group. While adolescents in the control group continued to receive usual education (at school or college), it may have been preferential to include an active control group or psychological placebo. Additionally, a cluster-randomised trial was not undertaken, as one site dropped out due to unforeseen circumstances. Implementing a cluster-randomised trial would reduce the likelihood of cross-contamination. A future trial could use an active control group and undertake cluster-randomisation, to help overcome these barriers. "

21) Generalisability (external validity, applicability) of the trial findings

NPT: External validity of the trial findings according to the intervention, comparators, patients, and care providers or centers involved in the trial

Your response is too large. Try shortening some answers.

21-i) Generalizability to other populations

Generalizability to other populations: In particular, discuss generalizability to a general Internet population, outside of a RCT setting, and general patient population, including applicability of the study results for other organizations

	1	2	3	4	5	
subitem not at all important	○	○	◉	○	○	essential

Clear selection

Does your paper address subitem 21-i?

Copy and paste relevant sections from the manuscript (include quotes in quotation marks "like this" to indicate direct quotes from your manuscript), or elaborate on this item by providing additional information not in the ms, or briefly explain why the item is not applicable/relevant for your study

This item is partly met in this paper as the paper discussed generalizability to a general Internet population - such as social media users, for example: "Given that adolescents favoured bite-sized education (eg., short weekly sessions), promoting short interactive digital communications via platforms such as SleepWise, could provide acceptable, time-efficient, and potentially efficacious solutions to encouraging and engaging adolescents with sleep behaviour change. Indeed, it is not surprising that online platforms such as social media are already being utilised to communicate health messages to adolescents (eg., see Yonker et al.51). To this end, it is clear that novel approaches to intervention development and delivery can help improve and maximise sleep health among adolescents, thus interventions like SleepWise warrant further exploration via a main trial of effectiveness.".

21-ii) Discuss if there were elements in the RCT that would be different in a routine application setting

Discuss if there were elements in the RCT that would be different in a routine application setting (e.g., prompts/reminders, more human involvement, training sessions or other co-interventions) and what impact the omission of these elements could have on use, adoption, or outcomes if the intervention is applied outside of a RCT setting.

	1	2	3	4	5	
subitem not at all important	○	○	◉	○	○	essential

Clear selection

Does your paper address subitem 21-ii?

Copy and paste relevant sections from the manuscript (include quotes in quotation marks "like this" to indicate direct quotes from your manuscript), or elaborate on this item by providing additional information not in the ms, or briefly explain why the item is not applicable/relevant for your study

This item is partly met in the limitation paragraph under the Discussion section: "Limitations of this study include the lack of an active control group. While adolescents in the control group continued to receive usual education (at school or college), it may have been preferential to include an active control group or psychological placebo. Additionally, a cluster-randomised trial was not undertaken, as one site dropped out due to unforeseen circumstances. Implementing a cluster-randomised trial would reduce the likelihood of cross-contamination.". Please see manuscript for further details.

OTHER INFORMATION

23) Registration number and name of trial registry

Does your paper address CONSORT subitem 23? *

Copy and paste relevant sections from the manuscript (include quotes in quotation marks "like this" to indicate direct quotes from your manuscript), or elaborate on this item by providing additional information not in the ms, or briefly explain why the item is not applicable/relevant for your study

Yes - this is provided in the Abstract page of this paper.

24) Where the full trial protocol can be accessed, if available

Does your paper address CONSORT subitem 24? *

Cite a Multimedia Appendix, other reference, or copy and paste relevant sections from the manuscript (include quotes in quotation marks "like this" to indicate direct quotes from your manuscript), or elaborate on this item by providing additional information not in the ms, or briefly explain why the item is not applicable/relevant for your study

Your response is too large. Try shortening some answers.

25) Sources of funding and other support (such as supply of drugs), role of funders

Does your paper address CONSORT subitem 25? *

Copy and paste relevant sections from the manuscript (include quotes in quotation marks "like this" to indicate direct quotes from your manuscript), or elaborate on this item by providing additional information not in the ms, or briefly explain why the item is not applicable/relevant for your study

Yes - this is provided under the "Acknowledgments" section of the paper.

X27) Conflicts of Interest (not a CONSORT item)

X27-i) State the relation of the study team towards the system being evaluated

In addition to the usual declaration of interests (financial or otherwise), also state the relation of the study team towards the system being evaluated, i.e., state if the authors/evaluators are distinct from or identical with the developers/sponsors of the intervention.

	1	2	3	4	5	
subitem not at all important	○	○	○	○	◉	essential

Your response is too large. Try shortening some answers.

Does your paper address subitem X27-i?

Copy and paste relevant sections from the manuscript (include quotes in quotation marks "like this" to indicate direct quotes from your manuscript), or elaborate on this item by providing additional information not in the ms, or briefly explain why the item is not applicable/relevant for your study

Yes - this is provided under the "Conflicts of Interest" section of this paper.

About the CONSORT EHEALTH checklist

As a result of using this checklist, did you make changes in your manuscript? *

- ○ yes, major changes
- ◉ yes, minor changes
- ○ no

What were the most important changes you made as a result of using this checklist?

Your response is too large. Try shortening some answers.

How much time did you spend on going through the checklist INCLUDING making changes in your manuscript *

It took me approximately four days to make sure the checklist was completed to a satisfactory standard and appropriate changes made to the manuscript.

As a result of using this checklist, do you think your manuscript has improved? *

- (x) yes
- ( ) no
- ( ) Other:

Would you like to become involved in the CONSORT EHEALTH group?

This would involve for example becoming involved in participating in a workshop and writing an "Explanation and Elaboration" document

- (x) yes
- ( ) no
- ( ) Other:

Your response is too large. Try shortening some answers.

Any other comments or questions on CONSORT EHEALTH

It's been an extremely useful exercise to go through this checklist to ensure my manuscript clearly communicates appropriate steps undertaken to carry out e-health research.

STOP - Save this form as PDF before you click submit

To generate a record that you filled in this form, we recommend to generate a PDF of this page (on a Mac, simply select "print" and then select "print as PDF") before you submit it.

When you submit your (revised) paper to JMIR, please upload the PDF as supplementary file.

Don't worry if some text in the textboxes is cut off, as we still have the complete information in our database. Thank you!

Final step: Click submit !

Click submit so we have your answers in our database!

Submit Clear form

Never submit passwords through Google Forms.

This content is neither created nor endorsed by Google. Report Abuse - Terms of Service - Privacy Policy

Your response is too large. Try shortening some answers.

Your response is too large. Try shortening some answers.
